# Twinkle twinkle brittle star: the draft genome of *Ophioderma brevispinum* (Echinodermata: Ophiuroidea) as a resource for regeneration research

**DOI:** 10.1186/s12864-022-08750-y

**Published:** 2022-08-11

**Authors:** Vladimir Mashanov, Denis Jacob Machado, Robert Reid, Cory Brouwer, Janice Kofsky, Daniel A. Janies

**Affiliations:** 1 Wake Forest Institute for Regenerative Medicine, 391 Technology Way, Winston-Salem, 27101 NC USA; 2 University of North Florida, Department of Biology, 1 UNF Drive, Jacksonville, 32224 FL USA; 3 University of North Carolina at Charlotte, College of Computing and Informatics, Department of Bioinformatics and Genomics, 9201 University City Blvd, Charlotte, 28223 NC USA; 4 University of North Carolina at Charlotte, College of Computing and Informatics, North Carolina Research Campus, 150 Research Campus Drive, Kannapolis, 28081 NC USA

**Keywords:** Echinodermata, Ophiuroidea, Brittle star, Tissue regeneration, Comparative genomics, Notch signaling pathway

## Abstract

**Background:**

Echinoderms are established models in experimental and developmental biology, however genomic resources are still lacking for many species. Here, we present the draft genome of *Ophioderma brevispinum*, an emerging model organism in the field of regenerative biology. This new genomic resource provides a reference for experimental studies of regenerative mechanisms.

**Results:**

We report a de novo nuclear genome assembly for the brittle star *O. brevispinum* and annotation facilitated by the transcriptome assembly. The final assembly is 2.68 Gb in length and contains 146,703 predicted protein-coding gene models. We also report a mitochondrial genome for this species, which is 15,831 bp in length, and contains 13 protein-coding, 22 tRNAs, and 2 rRNAs genes, respectively. In addition, 29 genes of the Notch signaling pathway are identified to illustrate the practical utility of the assembly for studies of regeneration.

**Conclusions:**

The sequenced and annotated genome of *O. brevispinum* presented here provides the first such resource for an ophiuroid model species. Considering the remarkable regenerative capacity of this species, this genome will be an essential resource in future research efforts on molecular mechanisms regulating regeneration.

**Supplementary Information:**

The online version contains supplementary material available at (10.1186/s12864-022-08750-y).

## Background

Echinoderms are a phylum of marine invertebrates, which together with hemichordates constitute the group Ambulacraria. In turn, the Ambulacraria form a sister clade to the phylum Chordata within the monophyletic superphylum Deuterostomia. Echinoderms have attracted much attention from scholars from various disciplines of biology (e.g., ecology, evolution, and developmental biology [1]). One of the fascinating aspects of the biology of many echinoderm species is the ability to regenerate all the tissues of large body parts and organ systems completely [2]. Thus far, attempts to understand the molecular underpinnings of echinoderm regeneration have been largely driven by transcriptome and gene expression studies [3–7]. Only recently functional perturbation studies started to emerge [3, 7–9], but the progress has been relatively slow in part due to the scarcity of genomic resources. Although the gene expression approaches are valuable in identifying the sets of genes that are involved in the regeneration program, gene expression studies do not by themselves lead to a comprehensive mechanistic understanding of regeneration. A mechanistic understanding of regeneration will be achieved through the reconstruction of the cause-and-effect relationships between regulatory and effector genes in functional genomics studies. The availability of genomic data is one of the essential prerequisites for these studies and it shall allow for the identification of *cis*-regulatory modules, reconstruction of chromatin accessibility maps, and the design of the genome editing experiments to probe for the function of candidate genes.

The efforts in echinoderm genomics were pioneered by the sequencing of the genome of the purple sea urchin, *Strongylocentrotus purpuratus* (Stimpson, 1857) [10]. Since then, the availability and affordability of the new generation high-throughput sequencing technologies have expanded genome sequencing and annotation projects to other species representing all five classes within the phylum. Table 1 lists the 21 echinoderm genomes that are currently available in public databases. These genomes, sequenced and annotated to a varying degree of completeness, have been submitted to the National Center for Biotechnology Information (NCBI) or other databases (e.g., [11–17]). These sequencing efforts, however, have rarely been driven by regeneration research. Only seven of the currently sequenced species have ever been studied in terms of their regenerative capacities (i.e., there is at least one published study on the topic). Only three of the sequenced species, the sea cucumbers *Holothuria glaberrima* and *Apostichopus japonicus* and the sea star *Patiria miniata*, are established model organisms in echinoderm regenerative biology (i.e., there has been continuous effort to characterize cellular and molecular events underlying regeneration). Two brittle star genomes are available at present, *Ophionereis fasciata* and *Ophiothrix spiculata*, but neither of those species has been used in studies of regenerative biology.

**Table 1:** Genome assembly statistics for echinoderm genomes available at the NCBI’s Assembly database (https://www.ncbi.nlm.nih.gov/assembly) on Jun. 17, 2021. Ditto marks (") indicate values identical to the cell above. Asterisks (*) indicate GenBank assembly accessions that have corresponding RefSeq assemblies

Organism name	Class: Order	GenBank assembly	Length (Mbp)	Scaffold No.	Scaffold N50 (bp)	Citations
*Asterias rubens*	Asteroidea:	GCA_902459465.3*	418	150	20,558,067	[81]
(European starfish)	Forcipulatida					
*Pisaster ochraceus*	"	GCA_010994315.1	402	1,844	20,188,303	[82]
(purple sea star)						
*Acanthaster planci*	Asteroidea:	GCA_001949145.1*	384	1,766	1,521,119	[16, 83, 84]
(crown-of-thorns starfish)	Valvatida					
*Patiria miniata*	"	GCA_000285935.1	811	60,183	52,614	NA
(bat star)						
*Patiriella regularis*	"	GCA_900067625.1	949	3,006,458	557	[17]
(starfish)						
*Anneissia japonica*	Crinoidea:	GCA_011630105.1*	590	76,727	623,489	NA
(crinoids)	Comatulida					
*Hemicentrotus pulcherrimus*	Echinoidea:	GCA_003118195.1	559	16,251	142,559	[12, 85]
(sea urchins)	Camarodonta					
*Lytechinus pictus*	"	GCA_015342785.1	999	1,307	46,003,000	[86]
(painted urchin)						
*Lytechinus variegatus*	"	GCA_018143015.1	870	104	45,600,000	[87],[88]
(green sea urchin)						
*Strongylocentrotus purpuratus*	"	GCA_000002235.4*	922	871	37,282,239	[10, 79, 89–106]
(purple sea urchin)						
*Eucidaris tribuloides*	Echinoidea:	GCA_001188425.1	2,187	637,071	39,192	NA
(sea urchins)	Cidaroida					
*Actinopyga echinites*	Holothuroidea:	GCA_010015985.1	899	895,374	1,907	NA
(sea cucumbers)	Holothuriida					
*Holothuria glaberrima*	"	GCA_009936505.1	1,128	346,783	1,221,172	NA
(sea cucumbers)						
*Apostichopus japonicus*	Holothuroidea:	GCA_002754855.1	805	3,278	487,241	[15]
(Japanese sea cucumber)	Synallactida					
*Apostichopus leukothele*	"	GCA_010014835.1	481	74,445	1,493,354	NA
(sea cucumbers)						
*Apostichopus parvimensis*	"	GCA_000934455.1	873	21,559	89,133	NA
(sea cucumbers)						
*Australostichopus mollis*	"	GCA_900067635.1	1,252	3,712,641	626	[17]
(sea cucumbers)						
*Paelopatides confundens*	"	GCA_011317855.1	1,379	764,445	504,687	NA
(sea cucumbers)						
*Stichopus horrens*	"	GCA_009801055.1	689	423,833	3,896	NA
(warty sea cucumber)						
*Ophionereis fasciata*	Ophiuroidea:	GCA_900067615.1	1,185	3,968,282	484	[17]
(brittle stars)	Amphilepidida					
*Ophiothrix spiculata*	"	GCA_000969725.1	2,764	75,696	72,780	NA
(brittle stars)						

**Fig. 1:**
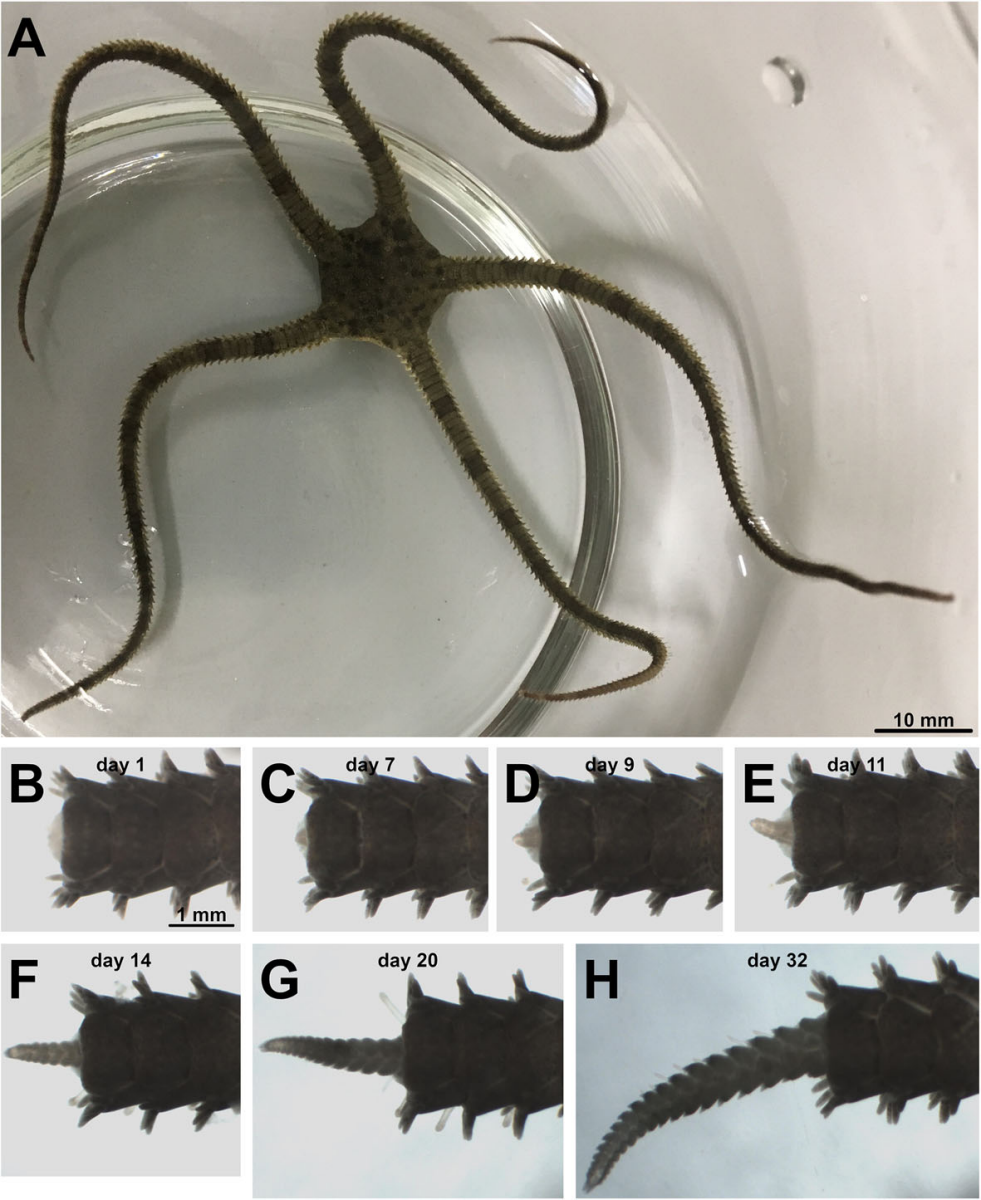
*Ophioderma brevispinum*, an emerging model organism in echinoderm regenerative biology. **A** An uninjured adult individual of *O. brevispinum*. **B**–**H** Regenerating arm at different time points post-injury. The regenerating distal end of the arm is to the left

The primary aim of this contribution is to provide genomic data and tools for a highly regenerative brittle star (ophiuroid echinoderm) *Ophioderma brevispinum* [18], an emerging model organism in the field of regenerative biology [3] (Fig. 1). This species is capable of autotomizing and quickly regenerating its arms, the segmented body appendages. Arm regeneration is a classic example of ”epimorphic” regeneration, which involves extensive unidirectional terminal growth through rapid generation of new tissues distal to the plane of the injury [3] (Fig. 1). As arms are often exposed to predators in natural habitats, brittle stars are known for their ability to sustain arm loss followed by remarkably high rates of arm regeneration. It has been estimated that regenerated tissues may account for as much as half of the total body weight in a brittle star individual [19]. It has also been shown that regeneration is temperature-dependent in at least some *Ophioderma* species [20].

The rationale for choosing this particular brittle star species for our research is as practical as it is fundamental. *O. brevispinum* is common in the Western Atlantic in shallow water and can be easily collected in numbers sufficient for molecular biology studies. During experiments, these animals are easily maintained in indoor aquaria and as research stock with minimal maintenance for extended periods (e.g., months). In addition, live individuals of *O. brevispinum* are available to all interested researchers, as they can be ordered from several commercial suppliers at a moderate price.

**Fig. 2:**
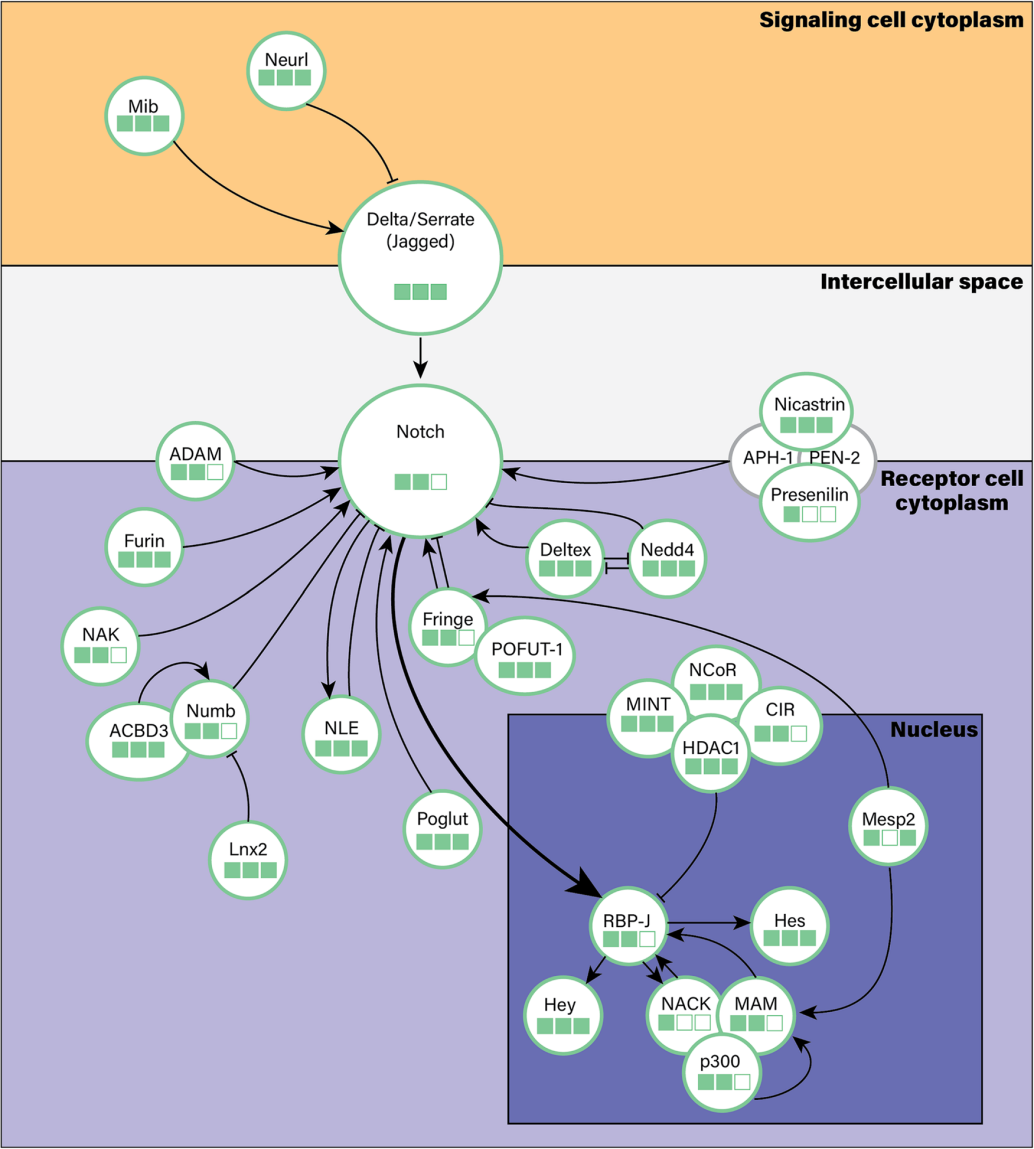
Simplified diagram of the Notch signaling pathway. The pathway is mediated by juxtacrine signaling that requires direct physical contact between the signaling and receptor cells. The *Delta/Serrate (Jagged)* ligands and *Notch* receptors are transmembrane proteins embedded into the plasma membrane of the signaling and receptor cells, respectively. Ligand-receptor interaction triggers conformational changes in the Notch protein that allows for proteolytic cleavage of the receptor by the *ADAM* metalloprotease and the multiprotein *γ*-secretase complex. The latter includes the catalytic component *presenilin*, as well as regulatory/stabilizing subunits *nicastrin*, *Aph-1*, and *Pen (presenilin enhancer)-2*. This proteolytic cleavage releases the Notch intercellular domain that translocates into the nucleus and activates the transcription factor *RBP-J* by inducing the release of co-repressors (e.g., *NCOR, CIR, MINT,* and *HDAC*) and recruitment of co-activators, such as *Mastermind (MAM), p300,* and *NACK*. The activated transcription factor complex initiates transcription of the direct targets of the pathway, including *Hes* and *Hey*. Even though the pathway itself is conceptually simple, it is subjected to a multitude of regulatory inputs at multiple levels, including receptor post-translational maturation and stability/availability of the key pathway components in both the signaling and receptor cells. One of the properties of the Notch pathway is the ability to sustain itself through a series of feed-forward loops, thus resulting in an all-or-nothing response. For example, *NACK*, which is a transcriptional co-activator in the pathway is itself positively regulated by Notch. The genes shown in the diagram were searched for and identified in the *O. brevispinum* draft genome (see Table 5). Three different searches for Notch related genes in the draft genome were performed, designated by green boxes (BLAST, Exonerate, and conserved domain search respectively), filled boxes indicate positive identification

Our previous work on *O. brevispinum* demonstrated a critical role of the Notch signaling pathway (Fig. 2) in ophiuroid arm regeneration [3]. This pathway is a key node of a complex hypernetwork of interconnected signaling pathways that mediate cell-cell interactions in various developmental contexts [21]. Thus a specific goal of this paper includes describing the genomic composition of the key components of the Notch pathway [21]. A report of these genes in *O. brevispinum* is provided to serve as a fundamental toolkit for future studies on regeneration. This work will facilitate further research to unravel the functions of signaling pathways in regeneration and identify their target genes through functional genomics approaches.

Here, we report a de novo genome assembly and annotation of *Ophioderma brevispinum*. This is the third ophiuroid for which genome sequencing has been applied following low coverage sequencing of *Ophiothrix spiculata* [14] (NCBI’s BioProject accession number PRJNA182997) and *Ophionereis fasciata* [17]. Our *Ophioderma brevispinum* genome and transcriptome [3] allow us to describe regulatory gene families to further explore the molecular bases of echinoderm regeneration. This use case is an example of one of the several applications for these genomic data that researchers will find.

## Results

### Sequencing

**Fig. 3:**
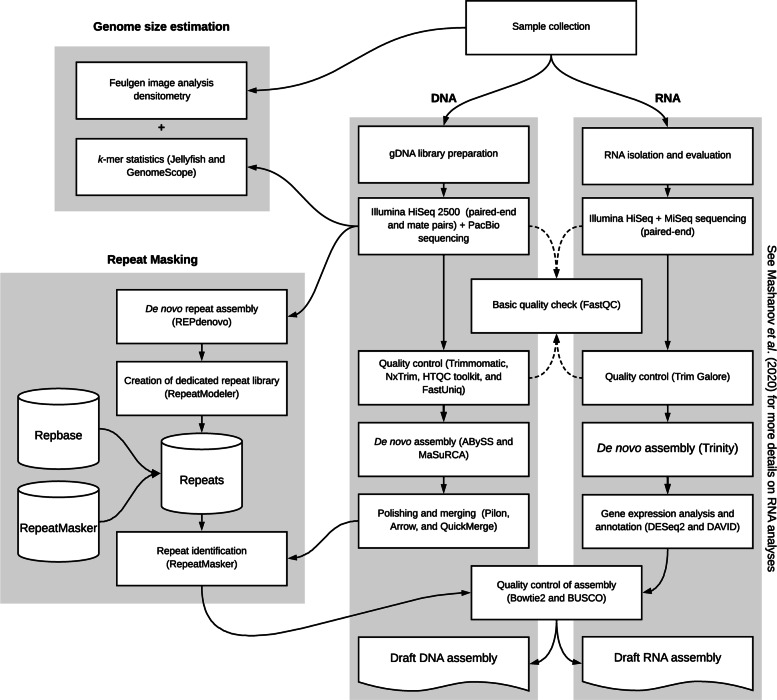
Schematic workflow of the *O. brevispinum* de novo DNA and RNA assembly. The main software tools used at each step of the workflow are shown in parenthesis. Grey boxes indicate four main components of the workflow. DNA: library preparation, quality control, high-throughput sequencing, and de novo assembly of gDNA. RNA: transcriptome assembly described in a previous publication. Genome size estimation: two different strategies used to estimate the haploid genome size. Repeat Masking: identification and categorization of repetitive DNA in the gDNA assembly. We used FastQC at different workflow steps to track the effect of quality control procedures on the sequence reads (see dashed arrowhead lines)

Three different genomic DNA library preparation and sequencing strategies were employed. First, PCR-free library preparation (“short” libraries) followed by sequencing on an Illumina HiSeq 25000 machine yielded 2 ×163,310,307 250 nt paired-end reads with an overall GC content of 37% (NCBI’s SRA accession number SRP238266). Second, mate-paired libraries (“long” libraries) with approximately 3 Kbp insert size were sequenced as above and resulted in 2 ×103,843,354 250 nt paired-end reads with an overall GC content of 41%. Third, PacBio long-read sequencing generated approximately 23 million reads with a total yield of 159 billion bp (51.3 × coverage) (Fig. 3). A summary of sequence read statistics is provided in Additional file 1: File S1.

In addition, in order to facilitate the annotation efforts, we took advantage of the earlier RNA-Seq study [3] that generated 17,318,775 MiSeq and 832,245,006 HiSeq quality filtered and adapter trimmed reads used in a de novo transcriptome assembly.

### Nuclear DNA assembly statistics

The draft assembly of the *O. brevispinum* genome generated 88,538 scaffolds with the total length of the assembly of 2,684,874,461 bp (approximately 2.68 Gb) (Table 2). This value is close to the haploid genome size independently determined by a densitometry assay (2.89 Gb) and from paired-end sequence data using a k-mer statistical approach (2.11 Gb). Scaffolds range from 2,035 bp to 612,917 bp, with a mean scaffold size of 30,325 bp. The N50 scaffold length and L50 scaffold size are 48,505 bp and 15,677, respectively. The scaffold nucleotide content is 30.77%, 19.22%, 19.18%, and 30.83% for A, C, G, and T, respectively.

**Table 2:** Summary metrics of the *O. brevispinum* genome assembly

Metrics	Quantification
Total assembly length	2,684,874,461 bp
Number of scaffolds	88,538
Shortest scaffold	2,035 bp
Longest scaffold	612,917 bp
Mean scaffold length	30,325 bp
N50 scaffold length	48,505 bp
L50 (scaffolds)	15,677
Assembly GC content	38.4%
Repetitive DNA	1,410,344,530 bp (52.53%)

An independent de novo assembly of repetitive DNA elements with REPdenovo resulted in 92,505 individual sequences with a total length of 134,023,983 bp. The average length and N50 of the repetitive DNA segments assembled this way were 1,448.83 bp and 2,838 bp (14,228 sequences), respectively. The sequences assembled with REPdenovo were used to aid in our draft genome assembly’s repeat identification and masking. A total of 1,410,344,530 bp (52.53%) of the draft assembly were classified as repetitive DNA and masked (see the summary of results in Table 3). Most DNA repeats (42.91% of the repetitive DNA sequence length) were classified as interspersed elements. However, a significant number of repeats (49.91% of the repetitive DNA) were marked as unclassified. The most common (5.27% of the repetitive DNA) transposable element (TE) in the classification of repeats corresponds to long interspersed nuclear elements (LINEs). Short interspersed nuclear elements (SINEs), repetitive DNA elements, and long terminal repeats (LTRs) amounted to 0.11%, 0.73%, and 1.12% of the total sequence length, respectively.

**Table 3:** Summary of results from RepeatMasker v4.0.8, run with rmblastn v2.6.0+. This table corresponds to the classification of 1,410,344,530 bp (GC content of 38.40%) of repetitive DNA in the draft genome assembly of *Ophioderma brevispinum*. (*) Most repeats fragmented by insertions or deletions have been counted as one element

	Number of elements (*)	Length occupied (bp)	Percentage of sequence
SINEs	20,483	2,893,927	0.11%
ALUs	0	0	0.00%
MIRs	20483	2893927	0.11%
LINEs	356,695	144,072,365	5.37%
LINE1	25	1,598	0.00%
LINE2	142,719	53,518,810	1.99%
L3/CR1	21,833	7,360,396	0.27%
LTR elements	44174	30,059,426	1.12%
ERVL	15	795	0.00%
ERVL-MaLRs	0	0	0.00%
ERV_classI	1	47	0.00%
ERV_classII	15	963	0.00%
DNA elements	68,647	19,627,664	0.73%
hAT-Charlie	295	13,023	0.00%
TcMar-Tigger	0	0	0.00%
Unclassified	4,673,979	1,143,432,629	42.59%
Total interspersed repeats		1,340,086,011	49.91%
Small RNA	0	0	0.00%
Satellites	219	83,876	0.00%
Simple repeats	644,098	68,988,714	2.57%
Low complexity	72,298	4,790,218	0.18%

In the GTF files from BRAKER and exonerate (Additional file 2: File S2), we found 361,060 exons in 146,703 genes, each corresponding to a different transcript in a total of 53,436 nuclear DNA scaffolds. According to position information, at least 3,394 of those 146,703 genes could represent gene isoforms. These putative isoforms are found in 1,311 scaffolds. The degree of fragmentation of this draft genome and limitations in resolving gene isoforms may partially account for this overestimation of the number of protein coding genes in *Ophioderma brevispinum*. These caveats should be addressed in future efforts to increase the completeness of this genomic resource.

The completeness of the draft genome assembly at the scaffold level was also evaluated with BUSCO [22, 23], a commonly used tool to assess the representation of marker genes in newly generated genomic and transcriptomic datasets. The results are summarized in Table 4. BUSCO analysis of this brittle star transcriptome has been previously reported elsewhere (see [3]).

**Table 4:** Summary of BUSCO v4.0.6 results for the scaffolds of the draft genome assembly. The database column names each odb10 BUSCO database used. The species column indicates the Augustus training parameter. Ditto marks (”) indicate values identical to the cell above. The names “Human”, “Fly” and “Spur” correspond to *Homo sapiens*, *Drosophila melanogaster*, and *Strongylocentrotus purpuratus*, respectively

Database	Species	Complete			Fragmented	Missing	N
		All	Single-copy	Duplicated			
Metazoa	Human	30.20%	29.60%	0.60%	16.00%	53.80%	954
”	Fly	30.10%	29.40%	0.70%	18.10%	51.80%	954
”	Spur	25.60%	25.30%	0.30%	19.70%	54.70%	954
Eukaryota	Human	21.6%	21.6%	0.00%	17.60%	60.80%	255
”	Fly	22.40%	22.00%	0.40%	15.30%	62.30%	255
”	Spur	20.00%	19.60%	0.40%	16.50%	63.50%	255

### Notch signaling pathway

To demonstrate the utility of the genome, we performed a case study in which we assessed the genomic representation of the main components of the Notch signaling pathway. This pathway is highly conserved across all multicellular animals and is known to coordinate a multitude of diverse cellular events, including: proliferation, differentiation, cell fate specification, and cell death [21, 24–29]. In the context of echinoderm regeneration, we have recently demonstrated that the proper function of the Notch pathway is crucial for the arm regeneration in *O. brevispinum* [3]. Here, we searched the draft genome for 29 genes involved in the pathway (Fig. 2, Table 5).

**Table 5:** Select components of the Notch signaling pathway identified in the draft genome of *O. brevispinum* using reference sequences from UniProt and Echinobase. For each gene, we list its name, the known function in the pathway, and whether or not the gene was recovered from the draft genome with independent BLAST and exonerate alignments. In addition, we also indicate if we could identify conserved protein domains in the predicted protein sequences

Name	Role in the Pathway	BLAST	Exonerate	Conserved domains	References
Delta/Serrate (Jagged)	Ligand of the Notch receptor	Yes	Yes	Yes	[21, 35]
Notch	Receptor	Yes	Yes	No	[21, 107]
RBP-J	Transcription factor activated by Notch	Yes	Yes	No	[21, 35]
Mastermind	Co-activator of RBP-J	Yes	Yes	No	[21, 35, 107]
p300	Co-activator of RBP-J	Yes	Yes	No	[108, 109]
NACK	Co-activator of RBP-J	Yes	No	No	[108, 110]
CIR1	Co-repressor of RBP-J	Yes	Yes	No	[21, 35]
NCoR	Co-repressor of RBP-J	Yes	Yes	Yes	[111]
HDAC1	Co-repressor of RBP-J	Yes	Yes	Yes	[112]
MINT	Co-repressor of RBP-J	Yes	Yes	Yes	[111]
Fringe	Post-translational maturation of Notch	Yes	Yes	No	[21, 35]
POFUT1	Post-translational maturation of Notch	Yes	Yes	Yes	[113]
Poglut	Post-translational maturation of Notch	Yes	Yes	Yes	[114]
Furin	Receptor proteolysis	Yes	Yes	Yes	[115]
Neuralized	Ubiquitination of Jagged	Yes	Yes	Yes	[3, 116]
Mindbomb	Ubiuitination of Delta	Yes	Yes	Yes	[117]
Nicastrin	Receptor proteolysis	Yes	Yes	Yes	[21, 118]
Presenilin 1	Receptor proteolysis	Yes	No	No	[21, 118]
ADAM 10/17	Metalloprotease	Yes	Yes	No	[21]
HES	Canonical target gene	Yes	Yes	Yes	[118–120]
HEY1	Canonical target gene	Yes	Yes	Yes	[118–120]
Numb	Negative regulator of the Notch pathway	Yes	Yes	No	[118]
LNX2	Negative regulator of Numb	Yes	Yes	Yes	[121]
ACBD3	Activator of Numb	Yes	Yes	Yes	[118, 122]
NAK	Positive regulator of the Notch pathway	Yes	Yes	No	[118]
Mesp2	Activates Fringe, induces degradation of Mastermind	Yes	No	Yes	[107]
Nedd4	Targets Notch and Deltex for degradation	Yes	Yes	Yes	[123]
Notchless	Context-dependent positive or negative regulator	Yes	Yes	Yes	[36]
Deltex	Context-dependent positive or negative regulator. Antagonizes Nedd4	Yes	Yes	Yes	[112, 123]

All genes were retrieved by BLAST [30] search. In addition, for all selected genes, except Mesp2, Presenilin 1, and NACK, we also recovered the same putative coding regions from exonerate alignments. In addition, in 18 genes, we also identified the expected conserved protein domains. Taken together, the newly assembled draft genome of *O. brevispinum* allowed us to retrieve the sequences of the Notch pathway components that will be subsequently used to design functional genomic studies to further probe into the mechanistic role of the pathway in brittle star regeneration.

### Mitogenome assembly

**Fig. 4:**
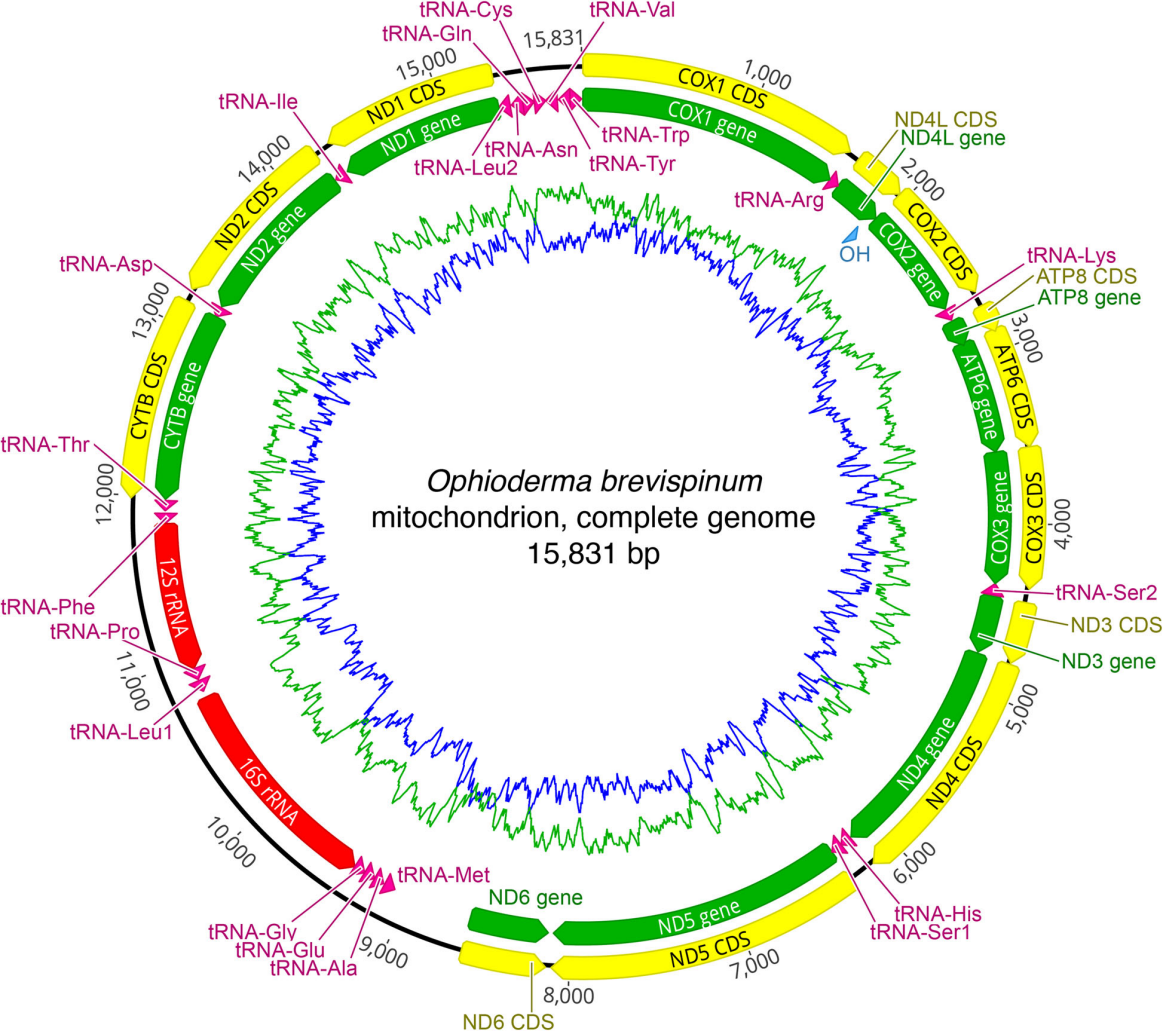
The complete mitochondrial genome of *Ophioderma brevispinum.* Arrows indicate the main genomic features and their orientation. The blue lines indicate variation in GC content. The green lines indicate variation in AT content

The *O. brevispinum* mitochondrial genome (mitogenome) is 15,831 bp long and has a GC content of 32.4% (Fig. 4). These values are similar to those of the previously published [31] reference mitogenome of another brittle star species *Ophiarachnella gorgonia* (NCBI accession number NC_046053), which has a length of 15,948 bp and a GC content of 36.7%. Likewise, mitochondrial genome features of *O. brevispinum* showed the same gene order reported for *O. gorgonia*, and their putative control regions are of similar length (488 and 474 bp, respectively).

There are also differences between these two brittle star mitogenomes that are worth noting. For example, the size difference between the mitogenomes of *O. brevispinum* and *O. gorgonia* is mostly due to deletions in non-coding intergenic regions. However, deletions in tRNA, rRNA, and protein-coding genes are also observed. Furthermore, unlike in *O. gorgonia*, the ND4 coding sequence in *O. brevispinum* is complete and does not add 3’ adenine residues to the mRNA.

## Discussion

Here, we present a draft genome assembly for the highly regenerative brittle star species *O. brevispinum*. Due to its availability, ease of maintenance, and amenability to experimental manipulations, *O. brevispinum* has become an emerging model organism in echinoderm regenerative biology [3]. We previously performed transcriptome-wide gene expression studies in this species and identified a range of candidate regeneration-associated genes for further experiments. However, without a fully sequenced genome, including non-coding and regulatory regions, it was not previously feasible to delve into the molecular mechanisms of regeneration with functional genomics tools for purposes such as reconstructing gene regulatory networks that underlie regenerative events. This draft genome of *O. brevispinum* provides the first such resource in ongoing and future molecular studies

The new genome has immediately allowed analysis of protein-coding genes. To demonstrate the utility of the genome, we aimed to retrieve 29 select components of the Notch signaling pathway, including the ligands, receptors, transcription factors, regulators, and target genes. All 29 genes of interest were identified in the assembly. The identity and predicted function of the proteins can be inferred by the presence of the conserved domains.

One of the limitations of our new draft genome assembly is its fragmented state. Ideally, the ultimate goal of any genome sequencing and annotation project is to reconstruct continuous chromosome-size sequences with the fully preserved order of the genes and non-coding sequence elements. Like many first-effort sequencing projects, our assembly will require subsequent efforts to reach that level. Even at its current state though, these data provide a valuable resource for the ongoing and future studies. This research will not be limited to regenerative biology, but can also benefit other areas, such as evolution of the echinoderm body plan, animal phylogeny, and history of gene families, to name a few.

## Conclusion

Here we presented the first draft nuclear genome and a complete mitochondrial genome of the brittle star *Ophioderma brevispinum* (Say, 1825) (Echinodermata: Ophiuroidea: Ophiacanthida: Ophiodermatidae), a rising model for regenerative studies (e.g., [3, 32–34]). The mitochondrial genome of this brittle star has 15,831 bp (with a mean depth of 1,658.7 and GC content of 32.4%) with 13 protein-coding genes, 22 tRNAs, and 2 rRNAs. The draft nuclear DNA assembly has 88,538 scaffolds summing up to 2.7 Gbp, corresponding to ∼93% of the expected haploid genome size independently determined by a densitometry assay. Despite the high degree of fragmentation of the assembly, which is partially caused by a high frequency of repetitive DNA elements (∼52.5% of the assembly), we demonstrated the usefulness of these data for biological investigation by identifying 29 key genes of the Notch signaling pathway, which is essential to tissue regeneration (e.g., [3, 26, 29, 35, 36]). We predict that the resources we are making available in this publication will be fundamental towards assembling the entire genome of *O. brevispinum* at the chromosomal level and establishing this brittle star species as a model for studies of regeneration and other fields.

## Methods

### Supporting genomic resources

Comparative genomic analysis relied on our original data and other genomic resources that are publicly available, including the complete genome of the purple sea urchin *Strongylocentrotus purpuratus* [10, 11] and other genomes available during the preparation of the manuscript (Table 1). These genomes represent 19 species and 17 genera of the classes Asteroidea (orders Forcipulatida and Valvatida), Crinoidea (order Comatulida), Echinoidea (orders Camarodonta and Cidaroida), Holothuroidea (orders Holothuriida and Synallactida), and Ophiuroidea (order Amphilepidida).

Additional interrogation and exploration of echinoderm genomic data leverage on resources made available through the Echinobase (www.echinobase.org) [37]. Recent reviews of genomic resources for the study of echinoderm development and evolution are available elsewhere [1, 38].

### Computational resources

All analysis steps where performed using computer clusters (Red Hat Enterprise Linux 7.5 with 64 CPUs and 512 GB to 1.5 TB of memory) as well as high-memory machines (Red Hat Enterprise Linux 7.5 with 16 CPUs and 512 GB to 4 TB of memory) at the University of North Carolina at Charlotte.

### Animal collection

Adult individuals of the brittle star *O. brevispinum* were obtained from the Marine Biological Laboratory (Woods Hole, MA, USA). Specimens (catalog no. 1970) were received on April 13, 2016. Immediately after delivery, the package was opened and left overnight to slowly allow the seawater to warm up to room temperature. The animals were then kept in aquaria with aerated artificial seawater.

### RNA-Seq

Complete RNA-Seq analysis (from RNA sampling and isolation until sequencing, de novo transcriptome assembly, and gene expression analysis) was described in [3]. Results correspond to BioProject number PRJNA596798 and SRA accession number SRP238266, and were deposited to NCBI’s Gene Expression Omnibus (GSE142391, https://www.ncbi.nlm.nih.gov/geo/query/acc.cgi?acc=GSE142391). BUSCO scores for the trasncritome assembly were presented in [3].

### DNA isolation and evaluation

A total of 100 mg of tissue was collected from the arms of a single non-regenerating adult individual through the natural autotomy response. The animal remained alive after tissue collection and regenerated its arms. The collected tissue samples were washed in filter-sterilized (0.2 *μ*m) seawater, cut into small pieces with a sterile blade, and put into the lysis buffer. The high molecular weight nuclear genome DNA was then extracted using the Qiagen MagAttract HMW kit according to the manufacturer’s instructions with the following modifications that were found to increase the yield and the molecular weight of the resulting DNA: 1) the vortexing speed with magnetic speed was reduced from 1,400 rpm to 1,200 rpm; and 2) after the first vortexing step with the magnetic beads the samples were allowed to sit for 5 min at room temperature before being placed into the magnetic rack. The concentration of the extracted DNA was assessed using the Qubit dsDNA Broad Range Kit (ThermoFisher). The total amount of DNA was determined at ∼50 *μ*g. The integrity of the genomic DNA sample was verified by agarose (0.6%) gel electrophoresis (at 2 V/cm, 4 hours).

### DNA library preparation and sequencing

All DNA sequencing was performed at the David H. Murdock Research Institute (DHMRI; Kannapolis NC, USA). Two different technologies were employed to obtain short and long sequence reads from the high molecular weight genomic DNA (HWM gDNA) sample extracted as described above (Fig. 3).

For sequencing on the Illumina HiSeq2500 platform, subsamples of ≥8 *μ*g of HMW gDNA were used to produce short sequence reads. Two complementary strategies were used to generate ”short” and ”long” insert libraries, respectively. First, three PCR-free paired-end read libraries (“short” insert size) with a ∼450 bp fragment size were constructed using the TruSeq DNA PCR-free library preparation kit (catalog no. FC-121-3001; Illumina, USA). Second, three mate paired-end read libraries (“long” insert size) were generated using the Illumina Nextera Mate Pair Sample Preparation Kit with the insert size of ∼3 Kbp. The short and long libraries were combined onto their respective pools and sequenced in the Rapid Mode to produce 2 × 251 bp reads.

To generate long sequence reads (∼10 Kbp), we used the Pacific Biosciences Single Molecule Real-Time (SMRT) platform [39]. Based on the estimated genome size of ∼2.9 Gbp (see “Genome size estimate”, below), we aimed to generate a ∼50 × coverage in an effort to improve the assembly by reducing and closing gaps. Over 10 *μ*g of HWM gDNA with an average fragment length of ≥60 Kbp was used to produce four SMRTbell libraries. The libraries were generated using the SMRTbell Template Prep Kit 1.0 following the PacBio “ >20 Kbp Template Preparation Using BluePippin Size-Selection System (15-20 Kbp) for Sequel Systems” procedures and checklist (catalog no. 100-286-000-07; Pacific Biosciences, USA). Libraries were sequenced in a combined total of 35 SMRTcells.

### DNA assembly and descriptive statistics

We performed several quality preprocessing steps on the raw DNA sequence reads before the assembly. The overall quality of sequence data before and after each step was determined by FastQC. See the supplementary information for additional details on the DNA assembly, including the commands used to execute the computational analyses described below (Additional file 3: File S3).

The Illumina reads from the short insert library were processed with Trimmomatic v0.38 [40] to remove the adapters and low-quality bases at the ends of each read. Trimmomatic also scanned each read with a 4-base sliding window, cutting when the average quality per base dropped below 15. Reads shorter than 36 bp were discarded. The long insert library Illumina reads were processed with NxTrim v0.3.0-alpha [41] using default parameters to separate reads into four different categories according to the adapter position: mate pairs, unknown (which are mostly mate pairs), paired end, and single end sequence reads.

All the sequence files produced by Trimmomatic and NxTrim were then evaluated with the HTQC toolkit v0.90.8 [42] to produce quality stats per file (using ht-stat) and perform the final read trimming and filtering (with ht-trim and ht-filter, respectively). Finally, FastUniq v1.1 [43] was used to remove duplicates introduced by PCR amplification from paired short reads.

All cleaned Illumina reads were used as input for the de novo assembly with ABySS v2.11 [44] using *k*-mers ranging from 23 to 61 (with a steps of 2). The individual assemblies generated at each *k*-mer value were ranked using several metrics, including the number of sequences, total assembly length, L50, and N50 [45]. We then polished the resulting best assembly in Pilon v1.2.3 [46] to improve base calling and detect sequence variation.

The long PacBio sequence reads we assembled with MaSuRCA v3.2.7 [47]. The contigs generated this way were polished using Arrow v2.3.3 [48] and merged with the ABySS assembly using quickmerge v3be7287 [49].

Assembly stats were calculated using the “assembly-stats” [50] (developed at the Wellcome Sanger Institute) and “assemblathon_stats.pl” [51] (developed a the UC Davis Bioinformatics Core) tools. The completeness of protein-coding gene representation in the scaffolds of the draft genome assembly was assessed with BUSCO v4.0.6 [22] run in the “genome mode” against the evolutionary conserved metazoan gene set (metazoa_odb10, creation date: 2021-02-17, number of species: 65, number of BUSCOs: 954) and the conserved eukaryota gene set (eukaryota_odb10, creation date: 2020-09-10, number of species: 70, number of BUSCOs: 255). Since *O. brevispinum* is not listed among the available species available for Augustus training, we tested other three species: *Homo sapiens*, *Drosophila melanogaster*, and *S. purpuratus*.

### Genome size estimate

The Animal Genome Size database has only one entry for species of *Ophioderma*, *O. panamensis* [52]. The expected C-value variation in echinoderms is provided in Additional file 4: File S4. That entry indicates that the expected *C*-value for *O. panamensis* is ∼3.3 pg (∼3.23 Gbp) based on bulk fluorometric assay [53]. The genome size of *O. brevispinum* have never been determined before. We, therefore, estimated it using two complementary approaches, including Feulgen densitometry (FD) assay [54–56] and also from the sequence data.

For the FD assay, soft uncalcified tissues (stomach wall and podia) from a single individual were finely minced with a razor blade, fixed in methanol:acetic acid (3:1) for 10 min and squashed in a drop of 45% acetic acid on a gelatin-coated slide. The samples were then air-dried and post-fixed in methanol:formalin:acetic acid (85:10:15) for 24 hours. After rinsing in tap and distilled water, the samples were hydrolyzed in 5N HCl and stained for 2 hours in Schiff reagent. After brief washes in a 0.5% sodium metabisulfite solution and then in water, the slides were dehydrated in an ethanol series, air dried and mounted in the immersion oil. Microscopic images were then taken at a consistent light intensity in the green monochromatic channel. The optical density of the stained nuclei was measured in the Fiji/ImageJ software [57]. To convert the optical density relative units to the absolute values of DNA mass per nucleus, the following control samples with known DNA content were processed and quantified along with the *O. brevispinum* specimens: chicken erythrocyte nuclei, trout erythrocyte nuclei, triploid trout nuclei, and human (male) cheek epithelial cell nuclei.

In addition to the FD assay, we also estimated the haploid genome size of *O. brevispinum* from the paired-end sequence data in Jellyfish v2.2.4 [58] using a *k*-mer-based statistical approach. The histograms produced by Jellyfish were used to estimate the haploid genome size in GenomeScope [59].

Detailed protocols for genome size estimation based on FD and on *k*-mer-based statistics are provided in Additional file 5: File S5. The estimated genome size was evaluated considering the variation in haploid genome size among echinoderms (see Additional file 6: File S6).

### Assembly and classification of repetitive DNA elements

Previous to this study, the reports of observed haploid genome size in echinoderms varied over 8-fold. Haploid genome size ranged from 0.53 Gbp in the sea star *Dermasterias imbricata* to 4.3 Gbp in the sea cucumber *Thyonella gemmata* [52]. The largest haploid genomes in the subphylum Asterozoa belong to the order Ophiurida, *Ophioderma panamensis*, with 3.3 Gbp [52].

In addition to the whole-genome assembly described above, we also performed a stand-alone de novo assembly of repetitive DNA elements in the genome of *O. brevispinum* with REPdenovo v0.0 [60] following the protocol described in the supplementary information (Additional file 7: File S7).

In short, we used REPdenovo to assemble repeats directly from the cleaned paired-end and single-end short sequence reads that resulted from the quality control steps described above, using different *k*-mer sizes ranging from 25 to 50 with a step of 2.

The contigs assembled with REPdenovo were used as input to RepeatModeler v1.0.11 [61] to build a library of repetitive genomic elements in the genome of *O. brevispinum*. This resulting brittle star repeat library was then combined with repeat libraries from the 2018 version of Repbase [62–65] and RepeatMasker v4.0.8 [66]. Only unique entries were kept to generate a final custom repeat library. This custom repeat library was then used to screen the draft genome of *O. brevispinum* with RepeatMasker to identify interspersed repeats and low complexity DNA sequences. Finally, the RepeatMasker output was manually curated and written into a General Feature Format version 3 (GFF3) file [67]. The details of the repeat library preparation and repeat masking are provided in Additional file 8: File S8.

### Gene prediction and annotation

**Fig. 5:**
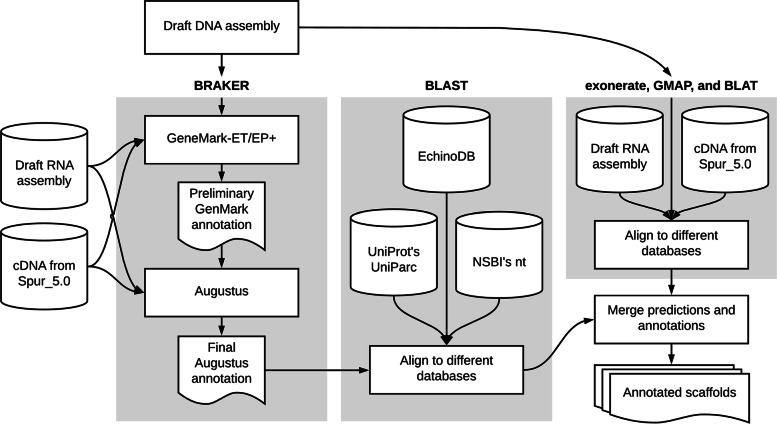
Schematic workflow of the procedures used for gene prediction and annotation of the *O. brevispinum* draft genome. Main steps (indicated by the grey boxes) are named according to the leading software used on each stage (BRAKER; BLAST; and exonerate, GMAP, and BLAT)

The gene prediction and annotation workflow is summarised in Fig. 5. We also provide template scripts listing the parameters used to execute each program listed below in Additional file 9: File S9.

Full gene structure annotations were generated with BRAKER v2.1.2 [68, 69], which integrates GeneMark-ET/EP+ v4.38 [70] and AUGUSTUS v.3.3.2 [71, 72] and allows for fully automated training from RNA-Seq or protein homology information. We also conducted an independent run with AUGUSTUS on selected scaffolds.

The BRAKER annotation pipeline used the genome of the purple sea urchin *S. purpuratus* (assembly Spur_5.0) as a reference [10, 73] and also the de novo assembled transcriptome of *O. brevispinum* [3].

The predicted gene models were aligned with BLAST v.2.9.0+ [30] against the following databases (each downloaded on October 25, 2019): the UniProt Archive (UniParc; https://www.uniprot.org/help/uniparc), the NCBI’s non-redundant nucleotide database (“nt”; https://ftp.ncbi.nlm.nih.gov/), and the complete EchinoDB database of protein coding genes (https://echinodb.uncc.edu/).

In addition, the genomic scaffolds were also aligned to the transcriptome of *O. brevispinum* and the cDNA sequences from *S. purpuratus* (assembly Spur_5.0) using exonerate v2.4.0ls [74], GMAP v2021.03.08 [75], and BLAT v36x2 [76].

The programs listed above generated different annotation tables. These tables were formatted as GTF files that are listed in the supplementary information. The main GTF files are provided in Additional file 2: File S2.

### Annotation of genes associated with the notch signaling pathway

As a case study, to demonstrate the practical utility of our draft genome assembly, we annotated selected core components and modifiers of the Notch signaling pathway (Fig. 2) using reference amino acid sequences from the UniProt and Echinobase (www.echinobase.org) [37] databases. The sequences from this query reference database were aligned to target exons from the BRAKER annotation using the TBLASTN program to search translated nucleotide databases (from scaffolds) using a protein query (described above) with the E-value, bit score, and percentage identity cutoff thresholds of 1.0E-5, 30.0, and 23%, respectively. In parallel, we also aligned the amino acids query sequences to all assembled scaffolds using exonerate to test if its exon predictions match BLAST results.

We used NCBI’s Conserved Domain Search (www.ncbi.nlm.nih.gov/Structure/cdd) to identify conserved protein domains in the brittle star Notch pathway genes returned by BLAST and/or exonerate. The conserved domains were searched against the CDD v3.19 database, with an E-value threshold of 0.01 and compositional-based statistics adjustment. We stored the best 500 hits for each gene sequence and then manually inspected the output for the presence or absence of diagnostic domains.

The complete list of genes related to the Notch signaling pathway we searched is provided in the Results section. Just as for the annotation tables for the genomic scaffolds, we also formatted the results of the annotation Notch-related genes in GTF files that we provide as supplementary information (Additional file 2: File S2).

### Mitochondrial genome

The mitochondrial genome (mitogenome) of *O. brevispinum* was contained in a single scaffold generated during the whole-genome assembly. It was identified via sequence alignments using BLAST v.2.9.0+ [30] and a reference sequence from an ophiuroid of the same family (NCBI’s accession number NC_046053.1), *Ophiarachnella gorgonia* (Müller & Troschel, 1842) (Echinodermata: Ophiuroidea: Ophiacanthida: Ophiodermatidae).

The putative circular sequence was extracted from the selected scaffold using AWA (available from https://gitlab.com/MachadoDJ/awa; accessed on July 22, 2021) [77]. Next, we remapped filtered short paired-end reads back to AWA’s putative mitogenome using Bowtie2 to review base calling. Finally, we used MITOS WebServer (version 2; available from http://mitos2.bioinf.uni-leipzig.de/index.py) [78] to predict genes and an independent analysis with tRNAscan-SE 2.0 [79, 80] to confirm the annotation of tRNAs.

## Supplementary Information

Supplementary information accompanies this paper at Zenodo, DOI: 10.5281/zenodo.6618000.

**Additional file 1** **File S1.** Summary of sequence read statistics (number of sequences, number of base pairs, maximum read length, average read length, sum, estimated genome size, and estimated overall sequence depth).

**Additional file 2** ∙ ‘notch-related.gtf‘: Independent annotation of genes that belong to the Notch signaling pathway. ∙ ‘repeatMasker.gtf‘: Repeat annotation based on similarity (not included in the gene statistics). ∙ ‘braker.gtf‘: Main gene annotation file produced with the BRAKER pipeline. ∙ ‘exonerate_complete.gtf‘: All hits from exonerate alignments (may include suboptimal hits because it stores the best hit per transcript query, not the best hit per target location). ∙ ‘exonerate_filtered.gtf‘: Filtered results from exonerate with the best hits per target location.

**Additional file 3** **File S3.** Bioinformatics protocols for quality control of raw sequence reads, subsequent genome assembly, and the methodology and main results for BUSCO v4.0.6 analyses.

**Additional file 4** **File S4.** Expected C-value variation in echinoderms. Data in this table is reference in our manuscript and is based mainly on information available from the “Animal genome size database” [52].

**Additional file 5** **File S5.** Protocols for Feulgen image analysis densitometry and sequence-based genome size estimation.

**Additional file 6** **File S6.** Expected variation of haploid genome sizes in echinoderms.

**Additional file 7** **File S7.** Protocol for de novo DNA repeat assembly from shotgun sequence reads.

**Additional file 8** **File S8.** Protocol for DNA repeat identification.

**Additional file 9** **File S9.** Template scripts for gene prediction and annotation.

## Data Availability

The data sets supporting the conclusions of this article are included within the article and its additional files. Supplementary information accompanies this paper is available at Zenodo, DOI: 10.5281/zenodo.6618000. Data corresponding to our draft genome assembly of *O. brevispinum* can be found at NCBI’s databases under BioProject number PRJNA779014, BioSample number SAMN23008116, and GenBank’s accession number JAMKCH000000000.1. The mitochondrial genome sequence and annotations have been submitted to NCBI under the same Bioproject number (PRJNA779014).

## Abbreviations

BAM: binary alignment map
BLAST: Basic Local Alignment Search Tool
bp: base pair
dsDNA: Double stranded DNA
EBI: European Bioinformatics Institute
EMBL: European Molecular Biology Laboratory
EMBOSS: European Molecular Biology Open Software Suite
ENCODE: Encyclopedia of DNA Elements
FASTA: it is pronounced “fast A” and stands for “Fast-All”
FASTP: it is pronounced “fast P” and stands for “Fast-Protein”
FD: Feulgen densitometry
FDR: false discovery rate
FPKM: fragments per kilobase of exon per million fragments mapped
GB: gigabyte (approx. 1024 MB)
Gb: same as Gbp
Gbp: giga base pairs (1,000,000,000 bp)
gDNA: genomic DNA
GFF3: general feature format or gene-finding format, version 3
GO: gene ontology
HTS: high-throughput sequencing
HMW: high molecular weight
InDel: insertion or deletion
Kb: same as Kbp
Kbp: kilo base pairs (1,000 bp)
L50: If we sort sequences by size and sum their sizes in succession from the shortest sequence, the L50 will be the number of sequences needed to achieve 50% of the total size
MB: a unit of information equal to 2^20^ bytes or, loosely, one million bytes
Mb: same as Mbp
Mbp: mega base pairs (1,000,000 bp)
mRNA: messenger RNA
MSA: multiple sequence alignment
mtDNA: mitochondrial DNA
nt: nucleotide
nucDNA: nuclear DNA
N50: If we sort sequences by size and sum their sizes in succession from the shortest sequence, the N50 will be the last sequenced added to achieve 50% of the total size
ORFs: open reading frames
PacBio: Pacific Biosciences
PE: paired-end (sequence of both ends of a fragment)
rRNA: ribosomal RNA
SMRT: Single Molecule Real-Time
SAM: sequence alignment/map
SE: standard errors
SR: single-read (sequencing from only one end)
ssDNA: single stranded DNA tRNA: transfer RNA
VCF: variant call file
