# Supplementary material for: Twinkle twinkle brittle star: the draft genome of Ophioderma brevispinum (Echinodermata: Ophiuroidea) as a resource for regeneration research
Source: BMC Genomics. 2022 Aug 11;23:574. doi: 10.1186/s12864-022-08750-y (PMC9367165; doi:10.1186/s12864-022-08750-y)
Supplement: Supplementary file 3 — Additional file 3 File S3. Bioinformatics protocols for quality control of raw sequence reads, subsequent genome assembly, and the methodology and main results for BUSCO v4.0.6 analyses. [file 12864_2022_8750_MOESM3_ESM.pdf]

Bioinformatics protocols for quality control and genome assembly

All original scripts are available athttp://www.ib.usp.br/grant/anfibios/researchSoftware.html and at https://gitlab.com/MachadoDJ under

the GNU General Public License version 3.0 (GPL-3.0).

Trimmomatic

We used Trimmomatic v0.38 to remove the adapters and low-quality bases at the ends of each read.

Variables:

- `TRIMMOMATIC_JAR`: Path to Trimmomatic
- `FILE1`: Path to the first paired-end sequence file
- `FILE2`: Path to the first paired-end sequence file

Main Bash command:

```
java -jar ${TRIMMOMATIC_JAR} \
    PE -phred33 \
    ${FILE1} ${FILE2} \
    trimmed_1_paired.fastq.gz trimmed_1_se.fastq.gz \
    trimmed_2_paired.fastq.gz trimmed_2_se.fastq.gz \
    ILLUMINACLIP:TruSeq3-PE-2.fa:2:30:10 \
    LEADING:3 TRAILING:3 SLIDINGWINDOW:4:15 MINLEN:36
```

NxTrim

Long insert size reads were processed in NxTrim v0.3.0-alpha using default parameters to separate reads into four different categories according to the adapter position: mate pairs, unknown (which are mostly mate pairs), paired-end, and single-end sequence reads.

NxTrim v0.3.0-alpha converts raw NMP reads into four "virtual libraries":

- **MP**: a set of known mate pairs having an outward-facing relative orientation and an effective genomic distance (EGD) whose distribution mirrors the size distribution of the circularized DNA
- **Unknown**: A set of read pairs for which the adapter could not be found within either read
- **PE**: a set of paired-end reads, having an inward-facing relative orientation and an EGD whose distribution mirrors the size distribution of the sequenced templates
- **SE**: a set of single reads

Variables:

- `FILE1`: Path to the first paired-end sequence file

- `FILE2`: Path to the first paired-end sequence file

Main Bash command:

```
nxtrim --separate -1 ${FILE1} -2 ${FILE2} -O nxtrimmed
```

HTQC toolkit

We employed the HTQC toolkit v0.90.8 to produce quality stats per tile (using `ht-stat`) and perform final read trimming and filtering (with `ht-trim` and `ht-filter`, respectively).

Variables:

- `PE1`: Path to the first input file of paired-end reads
- `PE2`: Path to the second input file of paired-end reads
- `SE1`: Path to the first input file of single-end reads
- `SE2`: Path to the first input file of single-end reads

Main Bash commands:

```
function part1 {
# STATS:
ht-stat -P -t 32 -z -i ${PE1} ${PE2} -o htstatpe > htstatpe_htqc.txt
wait
python2 selectTilesHTQC.py -d htstatpe > htstatpe_tiles.txt
wait
ht-stat -S -t 32 -z -i ${SE1} -o htstatse1 > htstatse1_htqc.txt
wait
python2 selectTilesHTQC.py -d htstatse1 > htstatse1_tiles.txt
wait
ht-stat -S -t 32 -z -i ${SE2} -o htstatse2 > htstatse2_htqc.txt
wait
python2 selectTilesHTQC.py -d htstatse2 > htstatse2_tiles.txt
wait
# TRIM:
ht-trim -z -i ${PE1} -o trimmed_pe1.fastq.gz &
ht-trim -z -i ${PE2} -o trimmed_pe2.fastq.gz &
ht-trim -z -i ${SE1} -o trimmed_se1.fastq.gz &
ht-trim -z -i ${SE2} -o trimmed_se2.fastq.gz
wait
}

function part2 {
# SIEVE:
ht-filter -P --filter length -z -i trimmed_pe1.fastq.gz trimmed_pe2.fastq.gz -o filteredpe &
ht-filter -S --filter length -z -i trimmed_se1.fastq.gz -o filteredse1 &
ht-filter -S --filter length -z -i trimmed_se2.fastq.gz -o filteredse2
wait
}

part1
```

```
part1
part2
```

FastUniq

We used FastUniq v1.1 to remove duplicates introduced by PCR amplification from paired short reads.

Description of input files (`files.txt`):

```
filteredpe_1.fastq
filteredpe_2.fastq
```

Main Bash command:

```
fastuniq -i files.txt -t q -o fastuniq_pair1.fastq -p fastuniq_pair2.fastq -c 0
```

ABySS

Main Bash script:

```
for KMER in {23..61..2} ; do
    abyss-pe -C k${KMER} k=${KMER} np=8 name=xb_k${KMER} lib='pea' mp='mpb' \
    pea='pe1.fastq pe2.fastq' \
    mpb='mate1.fastq mate2.fastq' \
    se='single.fastq'
done
```

Notes:

- `pe`: paired-end reads (short insert size)
- `mate`: mate paired-end reads (long insert size)
- `single`: single-end reads (resulting from quality control)

Pilon

Pilon v1.2.3 is a software tool that can be used to automatically improve draft assemblies and find variation among strains, including large event detection.

Pilon requires a FASTA file of the genome as input along with one or more BAM files of reads aligned to the input FASTA file. Pilon uses read alignment analysis to identify inconsistencies between the input genome and the evidence in the reads.

The initial step is to create the aligned BAM file from your raw reads. To create the aligned file we used Bowtie2 v2.2.9.

```
bowtie2-build -f genome.fasta aligned
bowtie2 -f -x aligned raw_reads.fasta -S output.sam
```

- The file `genome.fasta` is the whole genome file or the scaffolds that are being aligned to.
- The word `aligned` is the prefix on the index files that will be created.
- `-x`: is the prefix for the index files of the genome/scaffolds
- `-U`: unaligned reads

- `-S` : output in SAM format

The SAM output file needs to be comverted convert to BAM, sorted, and then indexed (the index file is necessary for Pilon to work). To do this, we used samtools v1.6.

```
samtools view -S -b aligned_output.sam > aligned_output.bam
samtools sort aligned_output.bam -o sorted_output.bam
samtools index sorted_output.bam
```

Finally, to execute Pilon:

```
java -jar pilon-1.23.jar --genome genome.fasta --bam sorted_output.bam --output pilon.out
```

MaSuRCA

Preparing the configuration file

The following is the commented template for creating the MaSuRCA configuration file that we used to create `masurca_config.txt`:

```
DATA

#################
## Paired end ###
#################

# pair ends (forward and reverse) must be listed in the same PE variable.
PE= pa 300 35 /path/to/forward.fastq /path/to/reverse.fastq

### If you have multiple paired-end sequences then add them as shown below ###
PE= pb 300 35 /path/to/forward.fastq /path/to/reverse.fastq
PE= pc 300 35 /path/to/forward.fastq /path/to/reverse.fastq
PE= pd 300 35 /path/to/forward.fastq /path/to/reverse.fastq

#################################
## Jumping Pairs (mate paired) ##
#################################

# mate paired (forward and reverse) must be listed in the same JUMP variable.
JUMP= ma 3000 500 /path/to/forward.fastq /path/to/reverse.fastq

### If you have multiple mate-pair sequences then add them as shown below ###
JUMP= mb 5000 750 /path/to/forward.fastq /path/to/reverse.fastq
JUMP= mc 8000 1000 /path/to/forward.fastq /path/to/reverse.fastq
JUMP= md 10000 1500 /path/to/forward.fastq /path/to/reverse.fastq

############
## PacBio ##
############

PACBIO= /path/to/pacbio/data.fasta

END

PARAMETERS

```

```
# GRAPH_KMER_SIZE is k-mer size for deBruijn graph values between 25 and 101 are supported, auto
GRAPH_KMER_SIZE=auto

# Set USE_LINKING_MATES to 1 for Illumina-only assemblies and to 0 if you have 2x or more long (S
USE_LINKING_MATES=0

#ILLUMINA ONLY. Set this to 1 to use SOAPdenovo contigging/scaffolding module.  Assembly will be
SOAP_ASSEMBLY=0

# Hybrid Illumina paired end + Nanopore/PacBio assembly ONLY.  Set this to 1 to use Flye assemble

# FLYE_ASSEMBLY=0

# Set this to 1 if your Illumina jumping library reads are shorter than 100bp
EXTEND_JUMP_READS=0

# Specifies whether to run the assembly on the grid
USE_GRID=0

# Specifies grid engine to use SGE or SLURM

# GRID_ENGINE=SGE

# Specifies queue (for SGE) or partition (for SLURM) to use when running on the grid MANDATORY

# GRID_QUEUE=all.q

# Batch size in the amount of long read sequence for each batch on the grid

# GRID_BATCH_SIZE=500000000

#set to 0 (default) to do two passes of mega-reads for slower, but higher quality assembly, other

# MEGA_READS_ONE_PASS=0

# Use at most this much coverage by the longest Pacbio or Nanopore reads, discard the rest of the
LHE_COVERAGE=25

# LIMIT_JUMP_COVERAGE is useful if you have too many jumping library mates. Typically set it to 6
LIMIT_JUMP_COVERAGE = 300

# These are the additional parameters to Celera Assembler. Do not worry about performance, number
CA_PARAMETERS = ovlMerSize=87 cgwErrorRate=0.1 ovlMemory=7.5GB merOverlapperThreads=8 cgwErrorRat

# CABOG ASSEMBLY ONLY: whether to attempt to close gaps in scaffolds with Illumina  or long read
CLOSE_GAPS=1

# Minimum count k-mers used in error correction 1 means all k-mers are used.  one can increase to
KMER_COUNT_THRESHOLD = 1

# Auto-detected number of cpus to use
NUM_THREADS= 64

# This is mandatory jellyfish hash -- a safe value is estimated_genome_size*20
JF_SIZE=180000000000
```

```
# This specifies if we do (1) or do not (0) want to trim long runs of homopolymers (e.g. GGGGGGG
DO_HOMOPOLYMER_TRIM=0

END
```

Configuration file

This is a comment-free version of `masurca_config.txt`:

```
DATA
PE= aa 300 35 25JUNE2016HiSeq_Run_Sample_Ob_PCRFree_UNCC_Janies_ACTTGA_L002_R1_001.fastq.gz 25JUN
END
PARAMETERS
GRAPH_KMER_SIZE=auto
USE_LINKING_MATES=1
LIMIT_JUMP_COVERAGE = 300
CA_PARAMETERS = ovlMerSize=87 cgwErrorRate=0.1 ovlMemory=7.5GB merOverlapperThreads=8
KMER_COUNT_THRESHOLD = 2
NUM_THREADS= 64
JF_SIZE=3400000000
DO_HOMOPOLYMER_TRIM=0
END
```

Executing MaSuRCA on UNC Charlotte's high-memory Lunix cluster

The following **PBS execution script** was used to run MaSuRCA at UNC Charlotte's 4TB memory machine that is part of the HammerHead Linux cluster:

```
#!/bin/bash

#PBS -l nodes=1:ppn=64
#PBS -l mem=3904gb
#PBS -N masurca.ob
#PBS -j oe
#PBS -q hammerhead
#PBS -l feature=mem_4tb
#PBS -l walltime=700:00:00

IFS=$'\n'
set -eu

umask 007
module load masurca/3.2.7
module list
masurca masurca_config.txt
bash assemble.sh
```

Quickmerge

Quickmerge uses a simple concept to improve the contiguity of genome assemblies based on long molecule sequences, often with dramatic improvement. The program uses information from assemblies made with Illumina short reads and Pacific Biosciences or Oxford Nanopore long reads to improve assembly contiguity with long reads alone. This is counterintuitive because Illumina short reads are not typically considered to cover genomic regions, which PacBio and ONP long reads cannot. Read more at

https://github.com/mahulchak/quickmerge. Also, read more about different strategies on how to use quickmerge at https://github.com/mahulchak/quickmerge/wiki.

Template command line:

`$ nohup merge_wrapper.py -pre [OUTPUT PREFIX] [MULTIFASTA BEST ASSEMBLY] [MULTIFASTA FRAGMENTED ASSEMBLY] >[STDOUT] 2> [STDERR] &`

The merge_wrapper.py script comes with quickmerge. It may take several hours to complete.

Assembly stats

Accessing assembly statistics in a multifasta file is easy with the `assemblathon_stats.pl` Perl script, which only requires `FAlite.pm` (add its to the PERL5LIB environmental variable, or place it in the same directory as other Perl modules). The script is availavle at https://github.com/ucdavis-bioinformatics/assemblathon2-analysis.

BUSCO

The completeness of protein-coding gene representation in the assembly was accessed with BUSCO v4.0.6 run in the "genome mode" against the evolutionary conserved metazoan gene set (metazoaodb10, creation date: 2021-02-17, number of species: 65, number of BUSCOs: 954) and the conserved eukaryota gene set (eukaryotaodb10, creation date: 2020-09-10, number of species: 70, number of BUSCOs: 255).

Since *O. brevispinum* is not listed among the available species available for Augustus training, we tested other three species: *Homo sapiens*, *Drosophila melanogaster*, and *Strongylocentrotus purpuratus*.

This is an example of an Bash execution script:

```
#!/usr/bin/bash

export AUGUSTUS_CONFIG_PATH="/path/to/AUGUSTUS_33/config/"
export BUSCO_CONFIG_FILE="/path/to/busco/config.ini"

module load busco/4.0.6
cd /path/to/working/directory

busco -i /path/to/assembly.fasta -c 60 -o OutputPrefix -e 1e-03 -m genome
--config config.ini -l /path/to/database

exit
```

We also ran BUSCO v4.0.6 on the predicted transcripts extracted from the BRAKER2 annotation using BUSCO's "protein mode." A table summarizing all BUSCO's results is below.

Database	Species	Mode	Complete	Complete and single-copy	Complete and duplicated	Fragmented	Missing	Total
eukaryota_odb10	Fly	Genome	57 (22.35%)	56 (21.96%)	1 (0.39%)	39 (15.29%)	159 (62.35%)	255
eukaryota_odb10	Fly	Protein	15 (5.88%)	15 (5.88%)	0 (0.00%)	92 (36.08%)	148 (58.04%)	255
eukaryota_odb10	Human	Genome	55 (21.57%)	55 (21.57%)	0 (0.00%)	45 (17.65%)	155 (60.78%)	255
eukaryota_odb10	Human	Protein	15 (5.88%)	15 (5.88%)	0 (0.00%)	92 (36.08%)	148 (58.04%)	255
eukaryota_odb10	S. purpuratus	Genome	51 (20.00%)	50 (19.61%)	1 (0.39%)	42 (16.47%)	162 (63.53%)	255
eukaryota_odb10	S. purpuratus	Protein	15 (5.88%)	15 (5.88%)	0 (0.00%)	92 (36.08%)	148 (58.04%)	255
metazoa_odb10	Fly	Genome	287 (30.08%)	280 (29.35%)	7 (0.73%)	173 (18.13%)	494 (51.78%)	954
metazoa_odb10	Fly	Protein	101 (10.59%)	94 (9.85%)	7 (0.73%)	264 (27.67%)	589 (61.74%)	954
metazoa_odb10	Human	Genome	288 (30.19%)	282 (29.56%)	6 (0.63%)	153 (16.04%)	513 (53.77%)	954
metazoa_odb10	Human	Protein	101 (10.59%)	94 (9.85%)	7 (0.73%)	264 (27.67%)	589 (61.74%)	954
metazoa_odb10	S. purpuratus	Genome	244 (25.58%)	241 (25.26%)	3 (0.31%)	188 (19.71%)	522 (54.72%)	954
metazoa_odb10	S. purpuratus	Protein	101 (10.59%)	94 (9.85%)	7 (0.73%)	264 (27.67%)	589 (61.74%)	954
