# Supplementary material for: Twinkle twinkle brittle star: the draft genome of Ophioderma brevispinum (Echinodermata: Ophiuroidea) as a resource for regeneration research
Source: BMC Genomics. 2022 Aug 11;23:574. doi: 10.1186/s12864-022-08750-y (PMC9367165; doi:10.1186/s12864-022-08750-y)
Supplement: Supplementary file 5 — Additional file 5 File S5. Protocols for Feulgen image analysis densitometry and sequence-based genome size estimation. [file 12864_2022_8750_MOESM5_ESM.pdf]

Genome size estimation

Feulgen image analysis densitometry

Materials

Materials to order

The following materials are usually not available in a molecular biology lab and may need to be ordered.

Supply	Supplier	Catalog No.
Activated charcoal	Sigma	05120-250G
Basic Fuchsin	Sigma	857343-25G
BioSure Trout Erythrocyte Nuclei (5.15 pg)	DNA/nucleus	Biosure
BioSure Chicken Erythrocyte Nuclei (2.5 pg)	DNA/nucleus	Biosure
BioSure Zebrafish Erythrocyte Nuclei (3.5 pg DNA/nucleus)	Biosure	1028
Sodium metabisulfite ($Na_2S_2O_5$)	Sigma	31448-500G

CMFSS-EDTA, pH 7.4

Reagent	Concentration	Weight per 100 ml
NaCl	449 mM	2.62 g
$Na_2S_2O_4 \cdot 10H_2O$	33 mM	1.06 g
KCl	9 mM	0.07 g
$NaHCO_3$	2.5 mM	0.02 g
EDTA (disodium)	1 mM	200 μl 0.5 M stock
H_2O		100 mL

0.4% Collagenase Type I

5N HCl

215 ml concentrated HCl + 285 ml H_2O = 500 ml 5N HCl.

1N HCl

459.5 ml H_2O + 40.5 ml concentrated HCL = 500 ml 1N HCl.

0.1N HCl

9 ml H_2O + 1 ml 1N HCl = 10 ml 0.1N HCl.

Schiff reagent, 550 ml

1. Completely dissolve 5.5 g of basic fuchsin in 50 ml 1 N HCl
2. Completely dissolve 5 g of sodium metabisulfite ($Na_2S_2O_5$) in 500 ml dH_2O and add to the fuchsin solution

3. Cap tightly and store at RT in the dark for 72 hrs shaking occasionally (at least twice a day) to dissolve any pink precipitate. The solution will turn from dark purple to wine-red

4. Add 2.5 g of activated charcoal (i.e. 0.5 g per 100 ml), shake vigorously for 2 min then filter through No. 40 Whatman filter paper. The stain should be water clean, or with minor pink or yellow tinting

5. Store in a full, tightly capped bottle at 4°C. Exposure to light should be minimized. Bring to

RT before use. Discard if a white precipitate forms

0.5% Bisulfite solution (SO2 rinse water), 100 ml

1. Made fresh daily
2. Dissolve 0.5 g of sodium metabisulfite ($Na_2S_2O_5$) in 95 ml of dH_2O
3. Add 5 ml of 1N HCl

0.075M KCl

Dissolve 0.56 g KCl in 100 ml H_2O.

Feulgen densitometry

Preparing samples for staining

Standards

The following standards were purchased from BioSure: * Chichen erythrocyte nuclei (CEN), Cat. No. 1006 * Trout Erythrocyte Nuclei (TEN), Cat. No. 1007 * Triploid Trout Nuclei, Cat No. 1014 The standards come in drop bottles. Mix a bottle for ~10 seconds, put one drop on a gelatin covered slide and prepare a smear from a 40 μl drop. Air dry and proceed with the staining.

Smear preparation

1. Collect the coelomic fluid into a 50 ml centrifuge tube
2. In the case of the male gonad, disrupt gonadal tubules in a small volume of the filtered seawater
3. Spread the cells (from a 40 μl drop)
4. Air dry the slides
5. Dip them into cold methanol
6. Air dry again

Squash preparations

1. Finely cut the tissue into pieces measuring ~1 mm in each direction

2. Fix the tissue pieces in methanol:acetic acid (3:1) for 10 min

3. Squash the samples in a drop of 45% acetic acid on a gelatin-covered slide

4. Let the slides dry for a few minutes

5. To remove the coverslip, freeze the slides on dry ice and thaw them in absolute EtOH

6. Air dry the slides and store in the dark

Disaggregation of solid tissues into a single cell suspension

1. Using two razor blades/scalpels, finely dissociate the tissue in a Petri dish. The remaining pieces should be smaller than 3 mm in any direction
2. Resuspend the tissue fragments in 9 ml sterile seawater. Pass through a 70 μm cell strainer into a 50 ml tube to remove the cells that have dissociated from the tissue during the mincing
3. Centrifuge the liquid that passed through the cell strainer at 400 g for 7 min at 4ºC. Decant the supernatant and dislodge the pellet. Resuspend the pellet in 2 mL of sterile seawater and place on ice until use
4. Transfer the tissue that did not pass through the cell restrainer into a new 50 mL conical tube (or Petri dish) containing 0.4% collagenase type I and 350 U/ml DNase I (add 35 μl DNase I, Roche 10 U/μl per ml)
5. Incubate at 28ºC for 1 hour
6. Pass the tissue/enzyme mixture through a new 70 m cell strainer into the tube containing previously collected single cells
7. Wash the digested tissue and the tube/dish used during the digestion with sterile seawater to remove any remaining single cells
8. Repeat digestion of any remaining tissue that did not pass through the cell stainer

Staining, image capture, and measurements

NOTE: Stain the experimental and control slides simultaneously 1. Fix the air-dried smears/squashed tissues in 85 methanol:10 formalin:5 acetic acid (MFA) for 24 hours 2. Rinse in tepid running tap water for 10 min 3. Dip in distilled water 4. Hydrolysis in 5N HCl for 30 min 5. Dip in 0.1N HCl for 5-10s 6. Stain for 2 hours in Schiff reagent 7. Rinse 3x5 min in fresh bisulfite solution 8. Rinse in tepid running tap water for 10 min 9. Rinse 3x5 min in distilled water 10. Dehydrate in ascending ethanols (70%, 95%, 100%, 2x5min each) 11. Air dry 12. Mount in immersion oil 13. To image, do the following: 13.1. Exposure: 100 ms 13.2. Light intensity: the brightest spots (in the green monochromatic channel) should be ~190. 13.3. Save images as 8bit grayscale 14. In ImageJ (Fiji) do the following: 14.1. Open the image 14.2. Invert the image: Edit → Invert (background pixels should have intensities close to zero) 14.3. Measure the largest nucleus (in pixels) using the Line Tool 14.4. Subtract background (Process → Subtract → Background). The Rolling Ball Radius parameter should be greater than the largest nucleus (200

for CEN). 14.5. Open the ROI Manager (Analyze → Tools → ROI Manager) 14.6. Using the Freehand Selection tool, select the first nucleus, press t to add the ROI to the ROI Manager. Repeat the procedure until all nuclei are selected. Avoid obviously distorted and overlapping nuclei 14.7. In the ROI Manager window, hit the Measure button 14.8. In the Results window: File → Save As and save the results as an `.xls` file

Chromosome analysis

1. Dissolve colchicine 50 mg/ml in ethanol (i.e. 250 mg in 5 ml). Intracoelomically inject 2 μl of this solution per 1 g of the body weight of regenerating animals (so, the dosage is 100 μg/g). Wait for 5 hours and then dissect the animals. Alternatively, if working with gonads, mix 2 μl of the conchicine stock (50 mg/ml in ethanol) wit 1 ml of filtered seawater. Incubate gonads in this solution for 4 hours
2. Prepare 0.1% Trypsin solution in CMFSS (1 mg/ml)
3. Cut the tissue into small pieces and incubate them in Trypsin solution for 10 min (or until the tissue is dissolved) at RT.
4. Spin at 1,000 g for 5 min to pellet the cells
5. Discard the supernatant
6. Add slowly 1 ml of 0.075M KCl, while gently tapping the tube. Cells must be resuspended completely.
7. Incubate at RT for 10 min
8. Centrifuge 1,000 g for 10 min; discard most of the supernatant leaving a small volume to resuspend the cells
9. Resuspend the cells in the remaining volume of the supernatant
10. Add 1 ml freshly prepared methanol-acetic acid (3:1), pre-cooled to -20ºC and resuspend the cells; resuspend the cells thoroughly after the first few drops of the fixative. Add remaining solution slowly
11. Incubate at RT for 10 min
12. Centrifuge at 400g for 5 min, decant the supernatant
13. Repeat fixation in fresh methanol-acetic acid one more time
14. After the last change of methanol-acetic, resuspend the tissue pieces in the sufficient volume of the fixative so that the suspension appears opaque. At this stage, the cells can be stored in the fixative at -20ºC for several months.
15. Place clean slides in ice water.
16. Remove a chilled slide; shake off excess moisture and immediately spread 10 l aliquots of the suspension from a height of 50 cm (apply some thumb pressure if needed)
17. Leave the slides for a few minutes in a moist chamber
18. Air dry overnight at 37ºC.
19. Stain in 5% Giemsa (Sigma GS500) diluted in PBS (pH 6.8) for 10 min

20. Briefly rinse in H2O
21. Air dry
22. Rinse in xylene 2x5 min
23. Mount in DPX

Estimating genome size from sequencing data

In addition to the Feulgen image analysis densitometry, we also produced genome size estimations based on sequencing data. For that, we used *k*-mer analisis of paired-end sequence data on `Jellyfish` v2.2.4 (see https://www.cbcb.umd.edu/software/jellyfish/ and http://www.genome.umd.edu/jellyfish.html#Release).

The input data are uncompressed `.fastq` files with short paired-end sequence reads indicated by the variables `input1` and `input2`. We used the following `Bash` script (named `Jellyfish.sh`) to execute `Jellyfish` in a Linux cluster:

```
module load jellyfish/2.2.4
cat $input1 $input2 > combined_input.fastq
for kmerSize in 17 21 23 31 ; do
jellyfish count -C -m ${kmerSize} -s 10G -t 8 -o kmer.counts.${kmerSize}
combined_input.fastq
wait
done
```

The result of the above operation is piped to Jellyfish, that does the following: 1. Counts *k*-mers of the `kmerSize` (as indicated in argument `-m`) 2. Hash table size: 10 billion entries (as indicated in argument `-s`) 3. Number of threads: 8 (as indicated in argument `-t`) 4. Output prefix (as indicated in argument `-o`)

The job ran for approximately 1 hour per value of *k*.

The following script, named `Jellyfish_histo.sh` was used to converting the *k*-mer count results into a histogram:

```
module load jellyfish/2.2.4
for kmerSize in 17 21 23 31 ; do
jellyfish histo -t 8 kmer.counts.${kmerSize} > histo.kmer.counts.${kmerSi
ze}
wait
done
```

The script ran for approximately 3 min per value of *k*. The output files were used as input for the web-based app GenomeScope (http://qb.cshl.edu/genomescope/info.php), generating output files in `.html` format. GenomeScope did not reported the "Genome Haploid Length" for *k*-mer sizes of 21 nt, 23 nt, and 31 nt, although the "Genome Repeat Length” and ”Genome Unique Length ” numbers are reported.
