# Supplementary material for: Twinkle twinkle brittle star: the draft genome of Ophioderma brevispinum (Echinodermata: Ophiuroidea) as a resource for regeneration research
Source: BMC Genomics. 2022 Aug 11;23:574. doi: 10.1186/s12864-022-08750-y (PMC9367165; doi:10.1186/s12864-022-08750-y)
Supplement: Supplementary file 6 — Additional file 6 File S6. Expected variation of haploid genome sizes in echinoderms. [file 12864_2022_8750_MOESM6_ESM.pdf]

Expected variation in the haploid genome size of echinoderms

The observed haploid genome size in echinoderms varies over 8-fold from 0.53 Gbp in *Dermasterias imbricata* (a sea star) to 4.3 Gbp in *Thyonella gemmata* (a sea cucumber). The largest haploid genomes in the subphylum Asterozoa belong to the order Ophiurida, and it is most extensive in the brittle star *Ophioderma panamensis* (3.3 Gbp).

Estimated C-values for echinoderm species

The following table was compiled from data available at the Animal Genome Size Database (Gregory, 2020). The "C-value" means the "constant" (or "characteristic") value of haploid DNA content per nucleus, typically measured in picograms (1 pg is approx. 0.978 Gbp). The chromosome number for all the species listed below is not available.

Phylum	Subphylum	Class	Order	Family	Species	Common Name	C-value (pg)	Method	Cell Type	Standard species
Echinodermata	Asterozoa	Stelleroidea	Forcipulatida	Asteriidae	*Asterias forbesi*	Sea star	0.60	BFA	S	SP
Echinodermata	Asterozoa	Stelleroidea	Forcipulatida	Asteriidae	*Astrometis sertulifera*	Sea star	0.70	BFA	S	SP
Echinodermata	Asterozoa	Stelleroidea	Forcipulatida	Asteriidae	*Pisaster giganteus capitatus*	Sea star	0.58	BFA	S	SP
Echinodermata	Asterozoa	Stelleroidea	Forcipulatida	Asteriidae	*Pisaster giganteus giganteus*	Sea star	0.68	BFA	S	SP
Echinodermata	Asterozoa	Stelleroidea	Forcipulatida	Asteriidae	*Pycnopodia helianthoides*	Sea star	0.79	BFA	S	SP
Echinodermata	Asterozoa	Stelleroidea	Ophiurida	Ophiocomidae	*Ophiocoma echinata*	Serpent star	2.40	BFA	S	SP
Echinodermata	Asterozoa	Stelleroidea	Ophiurida	Ophiocomidae	*Ophiopteris papillosa*	Serpent star	2.30	BFA	S	SP
Echinodermata	Asterozoa	Stelleroidea	Ophiurida	Ophiodermatidae	*Ophioderma panamensis*	Serpent star	3.30	BFA	S	SP
Echinodermata	Asterozoa	Stelleroidea	Ophiurida	Ophiothrichidae	*Ophiothrix spinosum*	Serpent star	2.20	BFA	S	SP
Echinodermata	Asterozoa	Stelleroidea	Ophiurida	Ophiuridae	*Ophioplocus esmarki*	Serpent star	3.00	BFA	S	SP
Echinodermata	Asterozoa	Stelleroidea	Spinulosida	Asterinidae	*Patiria miniata*	Sea star	0.76	BFA	S	SP
Echinodermata	Asterozoa	Stelleroidea	Spinulosida	Echinasteridae	*Echinaster echinophorus*	Sea star	0.96	BFA	S	SP
Echinodermata	Asterozoa	Stelleroidea	Spinulosida	Echinasteridae	*Echinaster sepositus*	Red starfish	0.76	FCM	HE	MGP
Echinodermata	Asterozoa	Stelleroidea	Spinulosida	Echinasteridae	*Henricia leviuscula*	Sea star	0.85	BFA	S	SP
Echinodermata	Asterozoa	Stelleroidea	Spinulosida	Echinasteridae	*Henricia sanguinolenta*	Sea star	0.89	BFA	S	SP
Echinodermata	Asterozoa	Stelleroidea	Spinulosida	Poraniidae	*Dermasterias imbricata*	Sea star	0.54	BFA	S	SP

Echinodermata	Echinozoa	Echinoidea	Arbacioida	Arbaciidae	*Arbacia aequituberculata*	Sea urchin	0.67	NS	S	NS
Echinodermata	Echinozoa	Echinoidea	Arbacioida	Arbaciidae	*Arbacia punctulata*	Sea urchin	0.76	BFA	S	SP
Echinodermata	Echinozoa	Echinoidea	Arbacioida	Arbaciidae	*Arbacia* sp.	Sea urchin	0.79	BCA	S	NS
Echinodermata	Echinozoa	Echinoidea	Clypeasteroida	Dendrasteridae	*Dendraster excentricus*	Sand dollar	1.10	BFA	S	SP
Echinodermata	Echinozoa	Echinoidea	Clypeasteroida	Echinarachniidae	*Echinarachnius parma*	Sand dollar	1.10	BFA	S	SP
Echinodermata	Echinozoa	Echinoidea	Clypeasteroida	Mellitidae	*Mellita quinquiesperforata*	Sand dollar	0.98	BFA	S	SP
Echinodermata	Echinozoa	Echinoidea	Diadematoida	Diadematidae	*Centrostephanus coronatus*	Sea urchin	1.20	BFA	S	SP
Echinodermata	Echinozoa	Echinoidea	Echinoida	Echinidae	*Paracentrotus lividus*	Sea urchin	0.54	FCM	HE	MGP
Echinodermata	Echinozoa	Echinoidea	Echinoida	Echinidae	*Paracentrotus lividus*	Sea urchin	0.90	NS	S	NS
Echinodermata	Echinozoa	Echinoidea	Echinoida	Echinidae	*Sterechinus neumayeri*	Sea urchin	0.69	BFA	NS	NS
Echinodermata	Echinozoa	Echinoidea	Echinoida	Echinometridae	*Echinometra lacunter*	Sea urchin	0.86	BFA	S	SP
Echinodermata	Echinozoa	Echinoidea	Echinoida	Echinometridae	*Echinometra mathaei*	Sea urchin	0.89	BCA	S	HS
Echinodermata	Echinozoa	Echinoidea	Echinoida	Echinometridae	*Echinometra* sp.	Sea urchin	0.98	FD	S	GD
Echinodermata	Echinozoa	Echinoidea	Echinoida	Strongylocentrotidae	*Strongylocentrotus droebachiensis*	Sea urchin	0.90	BFA	S	SP
Echinodermata	Echinozoa	Echinoidea	Echinoida	Strongylocentrotidae	*Strongylocentrotus franciscanus*	Sea urchin	0.83	BFA	S	SP
Echinodermata	Echinozoa	Echinoidea	Echinoida	Strongylocentrotidae	*Strongylocentrotus purpuratus*	Sea urchin	0.89	BFA	S	NS
Echinodermata	Echinozoa	Echinoidea	Spatangoida	Loveniidae	*Lovenia cordiformis*	Heart urchin	1.30	BFA	S	SP
Echinodermata	Echinozoa	Echinoidea	Temnopleuroida	Toxopneustidae	*Lytechinus pictus*	Sea urchin	0.97	BFA	S	SP
Echinodermata	Echinozoa	Echinoidea	Temnopleuroida	Toxopneustidae	*Lytechinus* sp.	Sea urchin	0.90	NS	S	NS
Echinodermata	Echinozoa	Echinoidea	Temnopleuroida	Toxopneustidae	*Lytechinus variegatus*	Sea urchin	0.92	BFA	S	SP
Echinodermata	Echinozoa	Echinoidea	Temnopleuroida	Toxopneustidae	*Tripneustes esculentus*	Sea urchin	1.10	BFA	S	SP
Echinodermata	Echinozoa	Holothuroidea	Apodida	Synaptidae	*Leptosynapta tenuis*	Sea cucumber	1.80	BFA	S	SP
Echinodermata	Echinozoa	Holothuroidea	Aspidochirotida	Holothuriidae	*Holothuria floridana*	Florida sea cucumber	2.30	BFA	S	SP

Echinodermata	Echinozoa	Holothuroidea	Aspidochirotida	Stichopodidae	*Stichopus californicus*	Sea cucumber	0.79	BFA	S	SP
Echinodermata	Echinozoa	Holothuroidea	Aspidochirotida	Stichopodidae	*Stichopus diabole*	Sea cucumber	0.99	FD	S	GD
Echinodermata	Echinozoa	Holothuroidea	Dendrochirotida	Cucumariidae	*Cucumaria rubi*	Sea cucumber	3.10	BFA	S	SP
Echinodermata	Echinozoa	Holothuroidea	Dendrochirotida	Cucumariidae	*Thyone briareus*	Sea cucumber	1.70	BFA	S	SP
Echinodermata	Echinozoa	Holothuroidea	Dendrochirotida	Cucumariidae	*Thyone mexicana*	Sea cucumber	1.90	BFA	S	SP
Echinodermata	Echinozoa	Holothuroidea	Dendrochirotida	Cucumariidae	*Thyonella gemmata*	Sea cucumber	4.40	BFA	S	SP
Echinodermata	Echinozoa	Holothuroidea	Dendrochirotida	Psloidae	*Thyonepsolus nutriens*	Sea cucumber	2.30	BFA	S	SP
Echinodermata	Echinozoa	Holothuroidea	Dendrochirotida	Sclerodactylidae	*Eupentacta* sp.	Sea cucumber	2.00	BFA	S	SP
Echinodermata	Echinozoa	Holothuroidea	Molpadida	Molpadiidae	*Molpadia arenicola*	Sea cucumber	0.85	BFA	S	SP

Abbreviations

- BCA: Biochemical Assay
- BFA: Bulk Fluorometric Assay
- FCM: Flow Cytometry
- FD: Feulgen Densitometry
- GD: *Gallus domesticus* (chicken), C-value of 1.25 pg
- HE: Haemocytes
- MGP: *Mytilus galloprovincialis* (Mediterranean mussel), C-value of 1.92 pg
- NS: Not Specified
- S: Sperm
- SP: *Strongylocentrus purpuratus* (Pacific purple sea urchin), C-value of 0.89 pg

References

- Fafandel, M., N. Bihari, Smodlaka, and S. Ravlic (2008). Hemocytes/coelomocytes DNA content in five marine invertebrates: cell cycles and genome sizes. Biologia 63: 730-736.
- Gregory, T.R. (2020). Animal Genome Size Database. http://www.genomesize.com. Accessed on Nov. 6, 2020.
- Hinegardner, R. (1974b). Cellular DNA content of the Echinodermata. Comparative Biochemistry and Physiology 49B: 219-226.
- Hinegardner, R.T. (1961). The DNA content of isolated sea urchin egg nuclei. Experimental Cell Research 25: 341-347.
- Marsh, A., P.K.K. Leong, and D.T. Manahan (1999). Energy metabolism during embryonic development and larval growth of an Antarctic sea urchin. Journal of Experimental Biology 202: 2041-2050.
- Marshak, A. and C. Marshak (1953). Desoxyribonucleic acid in Arbacia eggs. Experimental Cell Research 5: 288-300.
- Mirsky, A.E. and H. Ris (1951). The desoxyribonucleic acid content of animal cells and its evolutionary significance. Journal of General Physiology 34: 451-462.
- White, M.J.D. (1961). The Chromosomes. John Wiley & Sons, New York.
