# Supplementary material for: Twinkle twinkle brittle star: the draft genome of Ophioderma brevispinum (Echinodermata: Ophiuroidea) as a resource for regeneration research
Source: BMC Genomics. 2022 Aug 11;23:574. doi: 10.1186/s12864-022-08750-y (PMC9367165; doi:10.1186/s12864-022-08750-y)
Supplement: Supplementary file 7 — Additional file 7 File S7. Protocol for de novo DNA repeat assembly from shotgun sequence reads. [file 12864_2022_8750_MOESM7_ESM.pdf]

De novo DNA repeat assembly from shotgun sequence reads

Background

We executed all the computational analyses described here at UNC Charlotte's Steelhead cluster (see details at https://urc.uncc.edu/research-clusters). Several modifications may be needed to execute it in different computers.

Input data consisted of the paired-end sequence reads of short insert sizes resulting from our quality control, as described in **Additional file 1: File S1**.

De novo assemblies were performed in REP*denovo* v0.0 (available from https://github.com/simoncchu/REPdenovo), which is designed for constructing repeats directly from sequence reads based on the idea of frequent *k*-mer assembly.

Preparing REP*denovo's* input file

REP*denovo* takes sequence reads in the FASTQ format (uncompressed or compressed in .fastq.gz format). A raw reads file which lists the path, mean, and standard derivation of the insert-size should be provided in the following format:

`read-file-path group mean-insert-size insert-size-standard-derivation`

For single-end reads, `group`, `mean-insert-size`, and `insert-size-standard-derivation` should be set to -1.

For paired-end reads, two file paths should be provided, for each pair, on separate and consecutive lines. The "group" number should be the same for these two lines.

The following are copies of the input files we used.

Contents of `config_pe.txt`:

```
filteredpe_1.fastq.gz 1 450 50
filteredpe_2.fastq.gz 1 450 50
```

Contents of `config_se.txt`:

```
filteredse.fastq.gz -1 -1 -1
```

Preparing REP*denovo*'s configuration file

REP*denovo* needs a configuration file, which tells REP*denovo* the necessary settings. Users can find one sample from the same folder in REPdenovo's GitHub page (available at https://github.com/Reedwarbler/REPdenovo).

The following is a copy of the configuration file we used, in which `/path/to` is a placeholder for the specific paths we used and are not showing.

```
MIN_REPEAT_FREQ 10
RANGE_ASM_FREQ_DEC 2
RANGE_ASM_FREQ_GAP 0.8
K_MIN 25
K_MAX 50
K_INC 2
READ_LENGTH 250
GENOME_LENGTH 2836200000
MIN_CONTIG_LENGTH 249
ASM_NODE_LENGTH_OFFSET -1
IS_DUPLICATE_REPEATS 0.85
COV_DIFF_CUTOFF 0.5
MIN_SUPPORT_PAIRS 20
MIN_FULLY_MAP_RATIO 0.2
TR_SIMILARITY 0.85
TREADS 24
BWA_PATH /path/to/bin/bwa
SAMTOOLS_PATH /path/to/bin/samtools
JELLYFISH_PATH /path/to/bin/
VELVET_PATH /path/to/bin/
REFINER_PATH /path/to/TERefiner_1
CONTIGS_MERGER_PATH /path/to/ContigsMerger
OUTPUT_FOLDER /path/to/output
VERBOSE 1
```

Executing REP*denovo*

REP*denovo* is executed in two steps. First, the contigs are built from input that. Then, if paired-end data was provided, the expected insert size can be used for scaffolding. Both steps use the `main.py` executable that comes with REP*denovo*, and the user only need to change the arguments on the command line.

We executed REP*denovo* with the following script (named `run_repdenovo.sh`):

```
#!/usr/bin/env bash
# Define functions
function loading {
    # Load modules and show modules' list
    module load repdenovo/0.0
    module list
    # Local variables
    export CONFI="config_pe.txt"
    export INPUT="input_pe.txt"
}
function assemblying {
    # Build the repeats with REPdenovo
    main.py -c Assembly -g ${CONFI} -r ${INPUT} > assembly.out 2> assembly.
    wait
}
function scaffolding {
    main.py -c Scaffolding -g ${CONFI} -r ${INPUT} > scaffolding.out 2> sca
    wait
}
# Execute functions and quit
loading
assemblying
scaffolding
exit
```

This is the command line to execute the script above:

```
nohup bash run_repdenovo.sh > stdout.txt 2> stderr.txt &
```
