# Supplementary material for: Twinkle twinkle brittle star: the draft genome of Ophioderma brevispinum (Echinodermata: Ophiuroidea) as a resource for regeneration research
Source: BMC Genomics. 2022 Aug 11;23:574. doi: 10.1186/s12864-022-08750-y (PMC9367165; doi:10.1186/s12864-022-08750-y)
Supplement: Supplementary file 8 — Additional file 8 File S8. Protocol for DNA repeat identification. [file 12864_2022_8750_MOESM8_ESM.pdf]

Protocol for DNA repeat classification

Dependencies

The following programs and their corresponding dependencies are needed:

- RMBlast v2.6.0, available at http://www.repeatmasker.org/RMBlast.html
- RepeatScout v1.0.5, available at https://github.com/mmcco/RepeatScout
- RepeatMasker v4.0.8, available at http://www.repeatmasker.org/
- RepeatModeler v1.0.11, available at http://www.repeatmasker.org/RepeatModeler/

We are assuming these programs will be available as different modules named according to the program's name and organized by version.

This protocol was designed for a Linux computer.

Creating a custom repeat library with RepeatModeler

The following Bash script was used to execute RepeatModeler.

```
set -o errexit
# Function to check the number or arguments and define local variables:
if [ "$#" != 4 ] ; then
    echo "Error 0: Illegal number of parameters. Expected 4, found $#."
    exit
else
    export WD=$1
    export FASTA=$2
    export NAME=$3
    export PROCS=$4
fi
# Function to check working directory and input files:
function preparations {
    if [ -d ${WD} ] ; then
        cd ${WD}
        echo "WD=${WD}"
    else
        echo "Error 1: Cannot change into working directory."
        exit
```

```
    fi
    if [ -f ${FASTA} ] ; then
        echo "FASTA=${WD}/${FASTA}"
    else
        echo "Error 2: Cannot find input file."
        exit
    fi
}
# Function to load necessary modules:
function moduleload {
    module load rmblast/2.6.0
    module load repeatscout/1.0.5
    module load repeatmasker/4.0.8
    module load repeatmodeler/1.0.11
    module list
}
function builddb {
    BuildDatabase -name ${NAME} -engine ncbi ${FASTA}
    wait
}
# Function to execute RepeatModeler:
function runrm {
    RepeatModeler -engine ncbi -pa ${PROCS} -database ${NAME}
    wait
}
# Execute functions:
preparations
moduleload
builddb
runrm
exit
```

The script above can be executed as `bash ${scriptName} ${workingDir} ${pathToFasta} ${nameOfDatabase} ${noOfProcessors}`, where `scriptName` is the script's name, `workingDir` is the path to the working directory, `pathToFasta` is the path to the genome assembly in FASTA format, `nameOfDatabase` is the databases' name, and `noOfProcessors` are the numper of processors to use.

Time to completion will vary depending on computational resources, but in the case of a diploid genome of the size of about 3 Gbp, it will take approximately 48 hours to complete a single run. RepeatModeler will output several files into a directory with the prefix `R`. Within that directory, the main results will be named `consensi.fa.classifie`. This file contains all identified repeats and will be needed in the next stage.

Converting the RepeatMasker's repeat library to FASTA

format

The FASTA file extracted from **embl** files with `buildRMLibFromEMBL.pl` (available at https://github.com/rmhubley/RepeatMasker/blob/master/util/buildRMLibFromEMBL.pl). Make sure the generated file is complete and includes all standard headers and sequences.

Combining the repeat libraries and running RepeatMasker

You can concatenate the FASTA files from different sources to build a concatenated database.

The following is an example Bash script to execute RepeatMasker given a final version of the repeat library named `library.fasta` and a file containing the genome assembly named `genome.fasta`:

```
module load rmblast/2.6.0 repeatmasker/4.0.8
module list
function main {
    # This will run RepeatMasker on slow sensitive mode:
    RepeatMasker \
        -lib library.fasta \
        -s -gff -pa 10 \
        genome.fasta
}
main
exit
```
