# Supplementary material for: Twinkle twinkle brittle star: the draft genome of Ophioderma brevispinum (Echinodermata: Ophiuroidea) as a resource for regeneration research
Source: BMC Genomics. 2022 Aug 11;23:574. doi: 10.1186/s12864-022-08750-y (PMC9367165; doi:10.1186/s12864-022-08750-y)
Supplement: Supplementary file 9 — Additional file 9 File S9. Template scripts for gene prediction and annotation. [file 12864_2022_8750_MOESM9_ESM.pdf]

Template scripts for gene prediction and annotation

List of programs

We list the main programs used for gene prediction and annotation below. The links were tested on June 2, 2021.

- BRAKER v2.1.2: https://github.com/Gaius-Augustus/BRAKER
- GeneMark-ET/EP+ v4.38: http://exon.gatech.edu/GeneMark/
- AUGUSTUS v.3.3.2: https://github.com/Gaius-Augustus/Augustus
- BLAST v.2.9.0+: ftp://ftp.ncbi.nlm.nih.gov/blast/executables/blast+
- exonerate v2.4.0ls: https://www.ebi.ac.uk/about/vertebrate-genomics/software/exonerate
- GMAP v2021.03.08: https://github.com/juliangehring/GMAP-GSNAP
- BLAT v36x2: http://hgdownload.soe.ucsc.edu/downloads.html#source_downloads

Reading the template scripts

Template scripts have placeholders indicated within smaller than (`<`) and greater than (`>`) signs. For example, you should replace `</path/to/working/directory>` with the path to your working directory. Other comments start with a pound sign (`#`).

The following scripts were tested on Red Hat Enterprise Linux 7.5 using the UNC Charlotte's research clusters (https://oneit.uncc.edu/urc/research-clusters).

BRAKER's PBS execution script

The following tremplate script was used to execute BRAKER using the PBS job scheduler: ```

!/bin/bash

PBS -l walltime=168:00:00

PBS -l nodes=1:ppn=64

PBS -l mem=2000GB

PBS -N

PBS -j oe

PBS -q

IFS=$'\n' set -eu umask 007 shopt -s nullglob

Define variables:

MD= AUGUSTUS*CONFIG= GENEMARK=gmes*petap SPECIES=strongylocentrotus*purpuratus REFERENCE= ALIGNMENT= TRANSCRIPTOME*EVIDENCE= THREADS=64

Change to the working directory:

cd ${MD}

The modules belowmay not be available in your system, but this can be uses as a list of dependencies require to run the command line below:

module load anaconda3 module load module load augustus/3.3.2 module load genemark/4.38

module load blast/2.5.0+ module load braker

BRAKER's command line:

braker.pl --AUGUSTUS*CONFIG*PATH=${AUGUSTUS*CONFIG*} --*GENEMARK*PATH=${GENEMARK} --species=${SPECIES} --genome=${REFERENCE} --bam=${ALIGNMENT} --hints=${TRANSCRIPTOME_EVIDENCE} --filterOutShort --cores ${THREADS} --overwrite --skipAllTraining ```

Note that the shebang of the script above assumes that `bash` is in `/bin/`.

BLAST's execution command

The following template command line whas used to creat BLAST's Bash scripts:

```
blastx -query </path/to/fasta> -db </name/of/database> -out </path/to/out
put/file> -num_threads <number of threads> -outfmt 6
```

Note that the command line above assumes that you have a database prepared with `makeblastdb`.

Mapping transcripts to genomic scaffolds

We mapped the scaffolds from the brittle star, *Ophioderma brevispinum*, transcriptome (from Mashanov et al. 2020; DOI: 10.1371/journal.pone.0232981) to its draft genome assembly.

We also mapped the cDNA from the purple sea urchin, *Strongylocentrotus purpuratus* (file `Strongylocentrotus_purpuratus.Spur_5.0.cdna.all.fa`, available from https://metazoa.ensembl.org/Strongylocentrotus_purpuratus), to the draft genome of the brittle star, *Ophioderma brevispinum*.

We used BLAT v. 36x2, Exonerate v2.4.0ls, and GMAP v2021-03-08, as described below.

BLAT's SLURM script

```
#! /bin/bash

#SBATCH --partition=<partition>
#SBATCH --job-name=<name>
#SBATCH --nodes=1
#SBATCH --ntasks-per-node=36
#SBATCH --mem=375gb
#SBATCH --time=24:00:00

echo "======================================================"
echo "Start Time  : $(date)"
echo "Submit Dir  : $SLURM_SUBMIT_DIR"
echo "Job ID/Name : $SLURM_JOBID / $SLURM_JOB_NAME"
echo "Node List   : $SLURM_JOB_NODELIST"
echo "Num Tasks   : $SLURM_NTASKS total [$SLURM_NNODES nodes @ $SLURM_CPU
S_ON_NODE CPUs/node]"
echo "======================================================"
echo ""

blat -t=dna -q=rna -mask=lower -out=blast8 </path/to/genome/data/in/fasta
> </path/to/cdna/data/in/fasta> <output>

echo ""
echo "======================================================"
echo "End Time   : $(date)"
echo "======================================================"
```

GMAP's SLURM script

Create the genome database

```
#! /bin/bash

#SBATCH --partition=<partition>
#SBATCH --job-name=<name>
#SBATCH --nodes=1
#SBATCH --ntasks-per-node=36
#SBATCH --mem=375gb
#SBATCH --time=24:00:00

echo "======================================================"
echo "Start Time  : $(date)"
echo "Submit Dir  : $SLURM_SUBMIT_DIR"
echo "Job ID/Name : $SLURM_JOBID / $SLURM_JOB_NAME"
echo "Node List   : $SLURM_JOB_NODELIST"
echo "Num Tasks   : $SLURM_NTASKS total [$SLURM_NNODES nodes @ $SLURM_CPUS_ON_NODE CPUs/node]"
echo "======================================================"
echo ""

gmap_build -t 34 -k 15 --dir </path/to/destination/directory> --genomedb <genome_name> </path/to/genome/fasta/file>

echo ""
echo "======================================================"
echo "End Time   : $(date)"
echo "======================================================"
```

Align

```
#! /bin/bash

#SBATCH --partition=<partition>
#SBATCH --job-name=<name>
#SBATCH --nodes=1
#SBATCH --ntasks-per-node=36
#SBATCH --mem=375gb
#SBATCH --time=24:00:00

echo "======================================================"
echo "Start Time  : $(date)"
echo "Submit Dir  : $SLURM_SUBMIT_DIR"
echo "Job ID/Name : $SLURM_JOBID / $SLURM_JOB_NAME"
echo "Node List   : $SLURM_JOB_NODELIST"
echo "Num Tasks   : $SLURM_NTASKS total [$SLURM_NNODES nodes @ $SLURM_CPUS_ON_NODE CPUs/node]"
echo "======================================================"
echo ""

# Note that the command below expects a genome database (see script above).

gmap \
    -D </path/to/the/working/directory> \
    -d </name/of/the/genome/database> \
    -t 32 \
    -k 15 \
    -B 4 \
    </path/to/cdna/data/in/fasta> > <output> 2> <error>

echo ""
echo "======================================================"
echo "End Time   : $(date)"
echo "======================================================"
```

Exonerate's SLURM script

```
#! /bin/bash

#SBATCH --partition=<partition>
#SBATCH --job-name=<name>
#SBATCH --nodes=1
#SBATCH --ntasks-per-node=<cpus>
#SBATCH --mem=1000gb
```

```
#SBATCH --time=200:00:00

echo "======================================================"
echo "Start Time  : $(date)"
echo "Submit Dir  : $SLURM_SUBMIT_DIR"
echo "Job ID/Name : $SLURM_JOBID / $SLURM_JOB_NAME"
echo "Node List   : $SLURM_JOB_NODELIST"
echo "Num Tasks   : $SLURM_NTASKS total [$SLURM_NNODES nodes @ $SLURM_CPUS_ON_NODE CPUs/node]"
echo "======================================================"
echo ""

module load exonerate/2.4.0
module list

export QR1=</path/to/fasta/file>
export QR2=</path/to/fasta/file>
export SCF=</path/to/fasta/file>
export WDR=</path/to/working/directory>

for i in {1..100} ; do
FILE="exonerate_Sp_${i}.out"
if [ -f "${FILE}" ]; then
printf "! ${FILE} exists (skipping).\n"
else
printf ":-) ${FILE} does not exist (exonerating).\n"
exonerate --percent 75 --dpmemory 500000 --fsmmemory 500000 --softmasktarget yes --verbose 1 --showalignment no --showvulgar no --showsugar no --showcigar no --showtargetgff yes --model est2genome --querychunkid ${i} --querychunktotal 100 ${QR2} ${SCF} > exonerate_Sp_${i}.out 2> exonerate_Sp_${i}.err
wait
fi
printf "\n//\n"
done

echo ""
echo "======================================================"
echo "End Time   : $(date)"
echo "======================================================"
```
